# Supplementary figures and images for: A precisely adjustable, variation-suppressed eukaryotic transcriptional controller to enable genetic discovery
Source: eLife. 2021 Aug 3;10:e69549. doi: 10.7554/eLife.69549 (PMC8421071; doi:10.7554/eLife.69549)

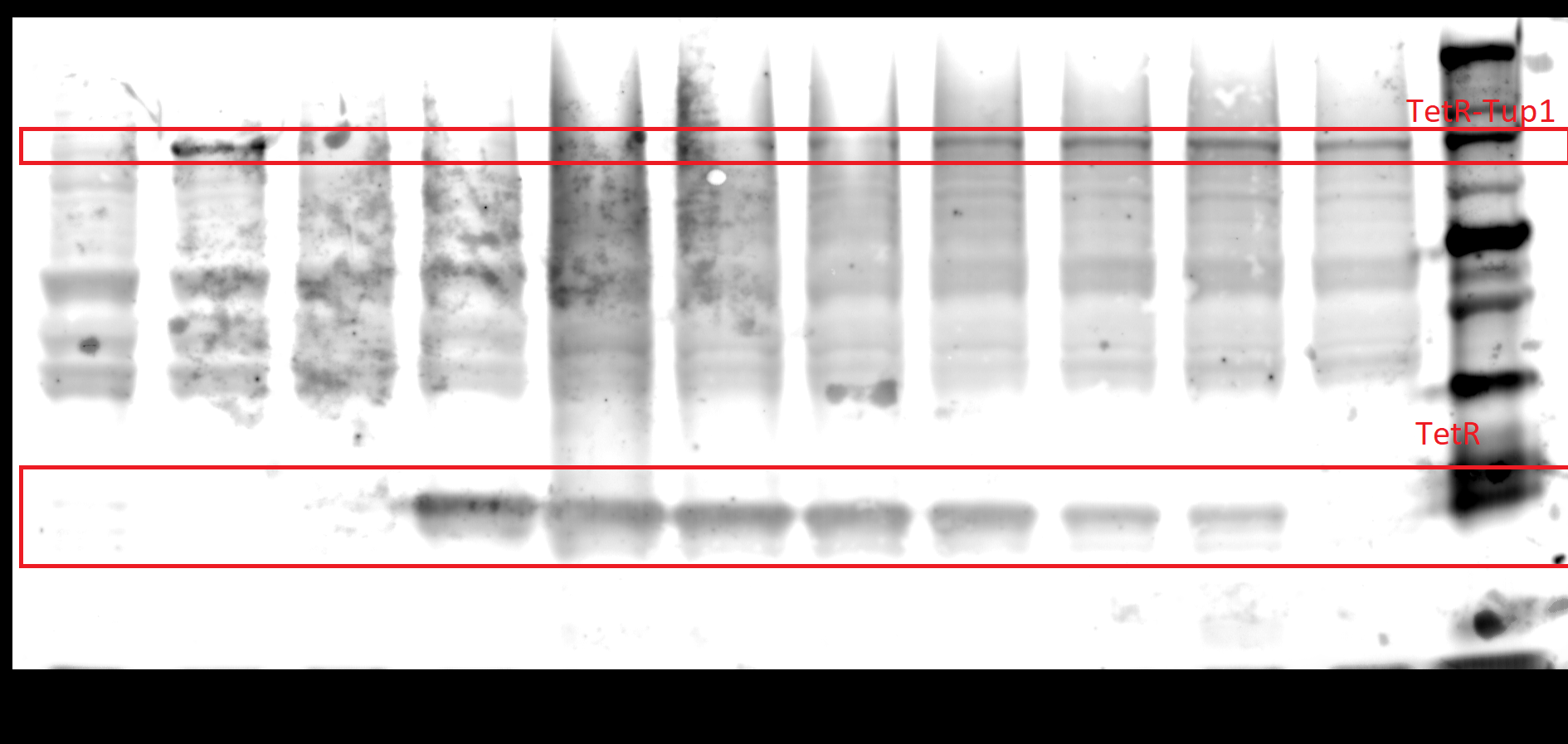

Supplement: Figure 4—figure supplement 1—source data 1. [file elife-69549-fig4-figsupp1-data1.zip › Figure4- figure supplement 1- source data 1/PanelA_tetR_doseR_modified.tif]

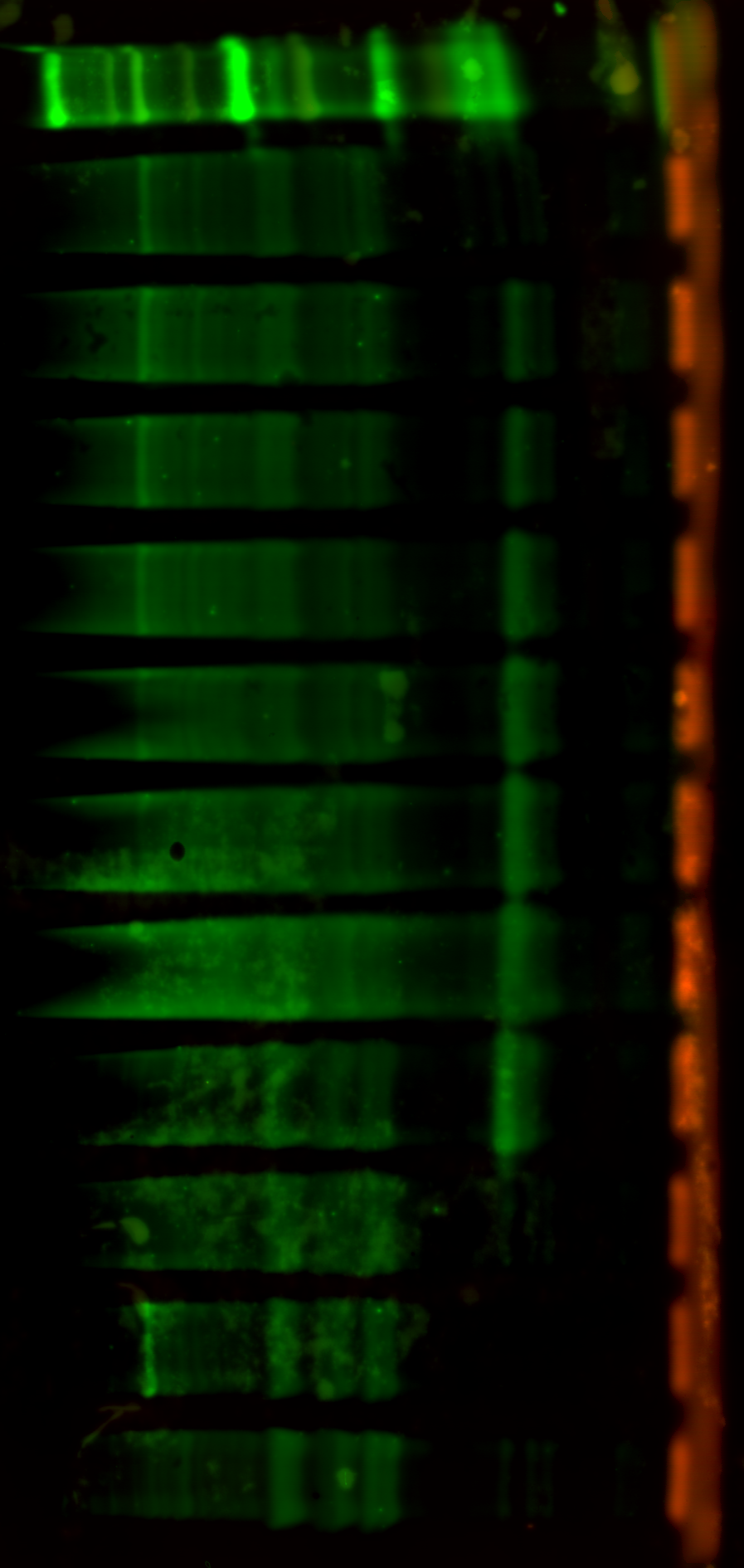

Supplement: Figure 4—figure supplement 1—source data 1. [file elife-69549-fig4-figsupp1-data1.zip › Figure4- figure supplement 1- source data 1/PanelA_tetR_doseR_raw.tif]

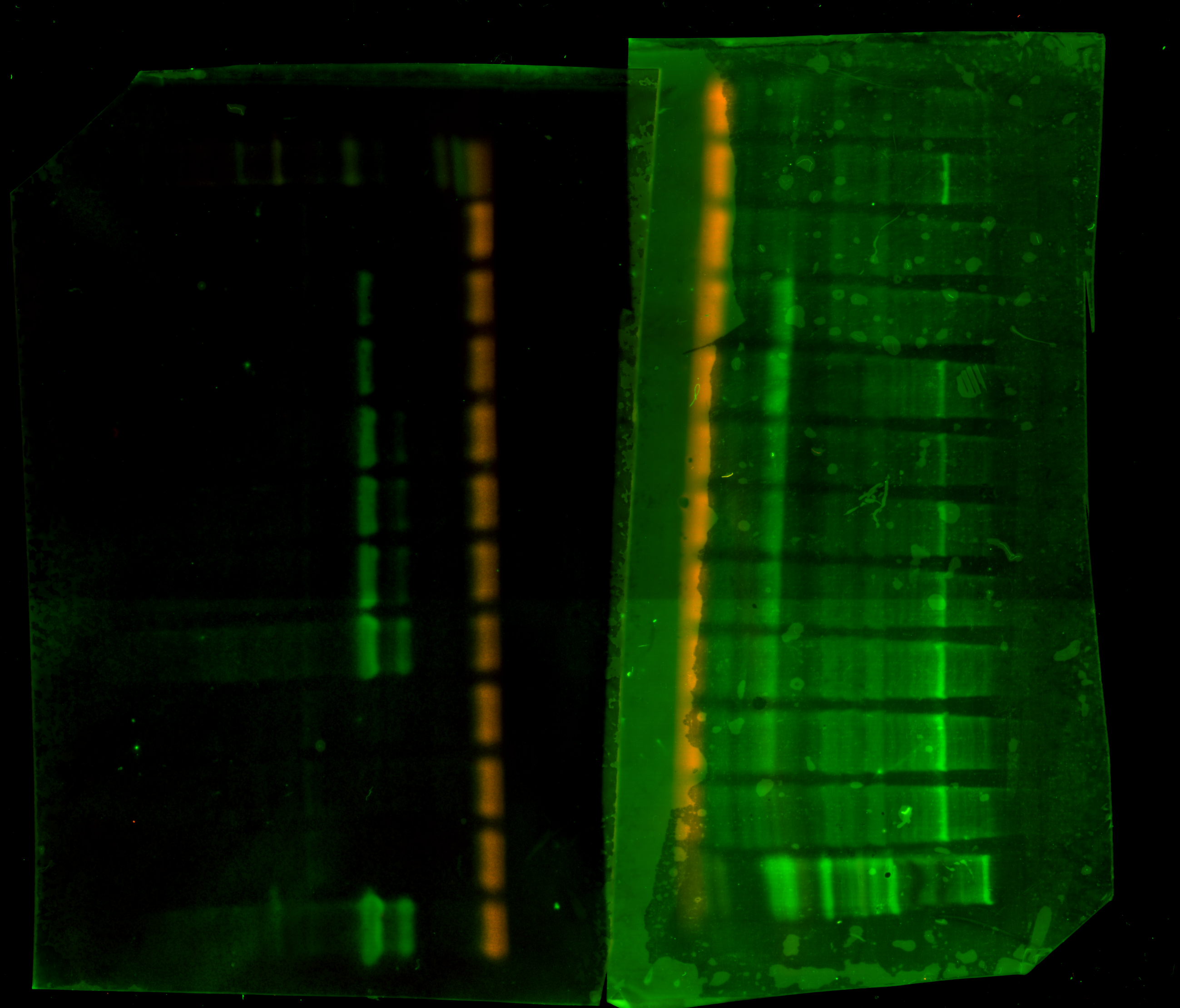

Supplement: Figure 4—figure supplement 1—source data 1. [file elife-69549-fig4-figsupp1-data1.zip › Figure4- figure supplement 1- source data 1/PanelB_citrine_doseR_top_raw.tif]

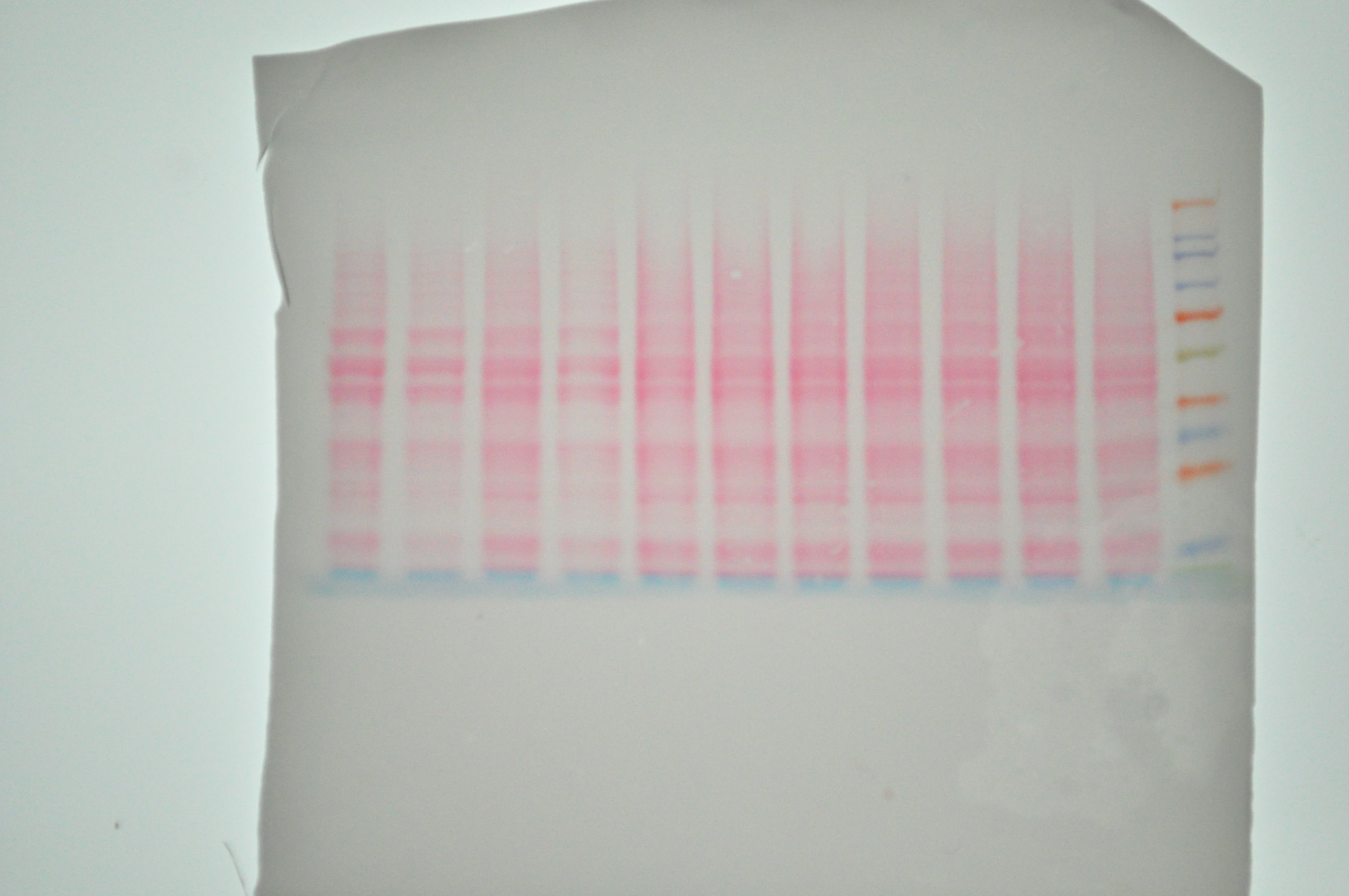

Supplement: Figure 4—figure supplement 1—source data 1. [file elife-69549-fig4-figsupp1-data1.zip › Figure4- figure supplement 1- source data 1/PanelC_TetR_doseR_ponceau_raw.JPG]

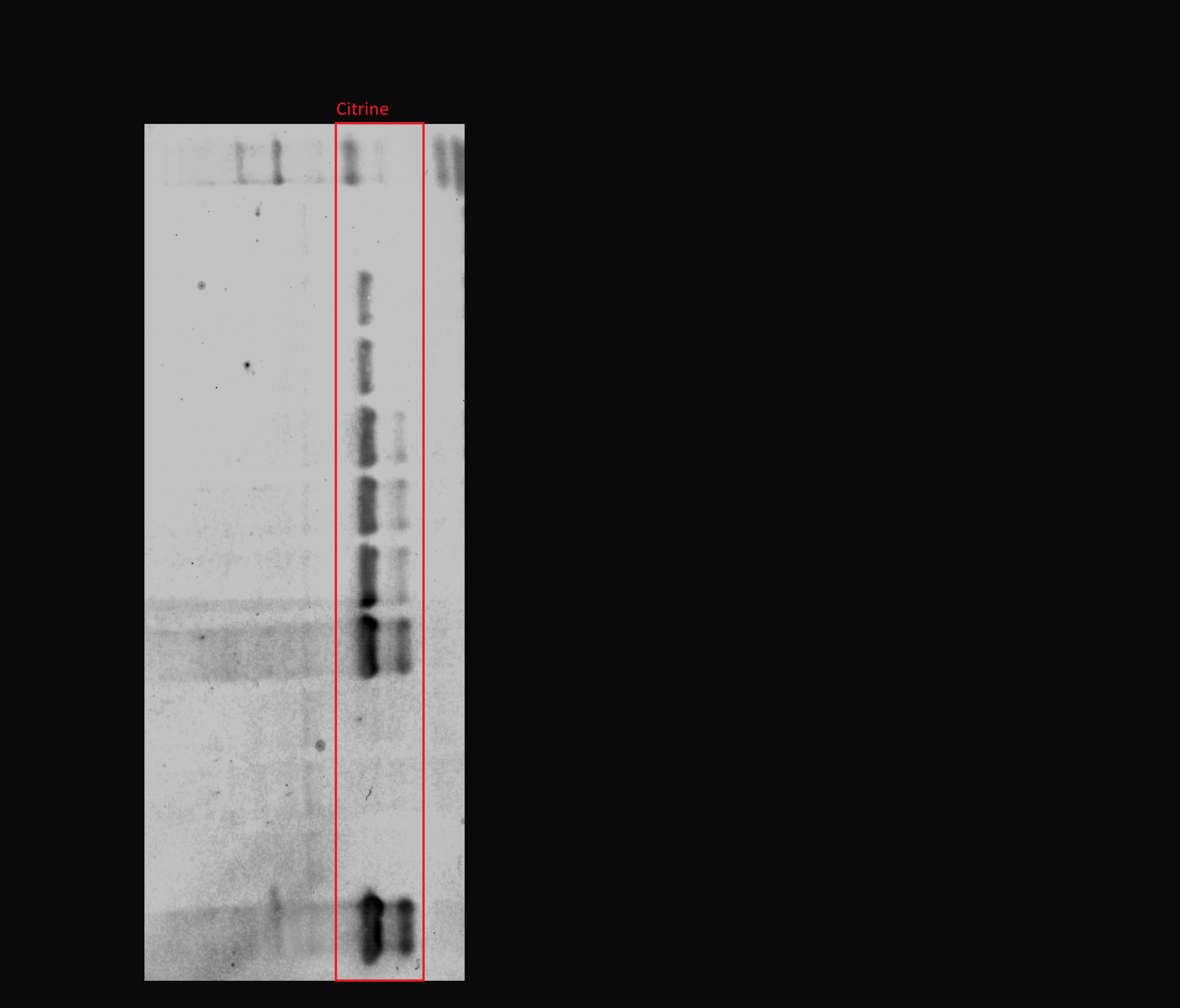

Supplement: Figure 4—figure supplement 1—source data 1. [file elife-69549-fig4-figsupp1-data1.zip › Figure4- figure supplement 1- source data 1/PanelD_citrine_doseR_modified.tif]

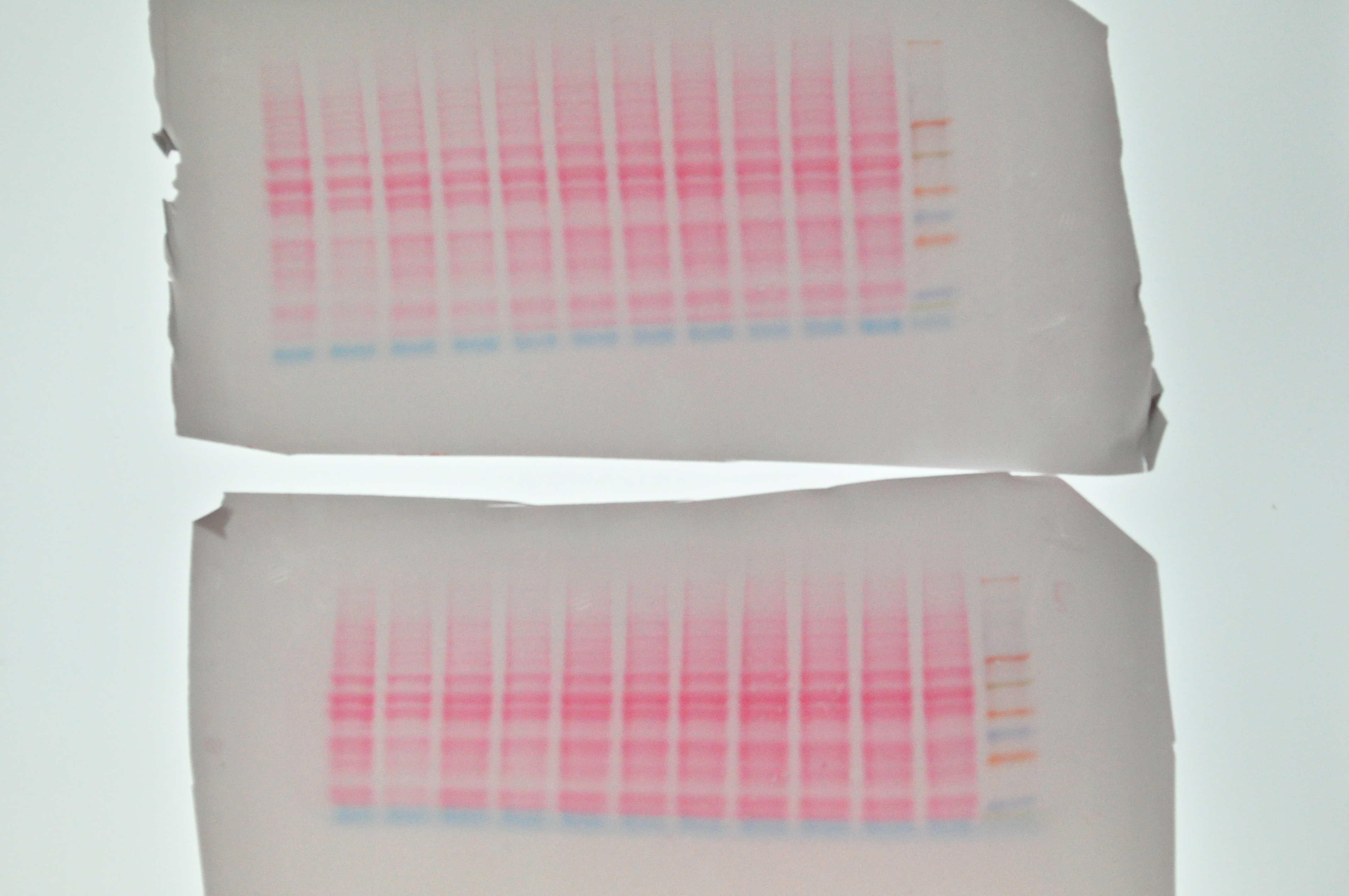

Supplement: Figure 4—figure supplement 1—source data 1. [file elife-69549-fig4-figsupp1-data1.zip › Figure4- figure supplement 1- source data 1/PanelD_citrine_doseR_ponceau_raw.JPG]

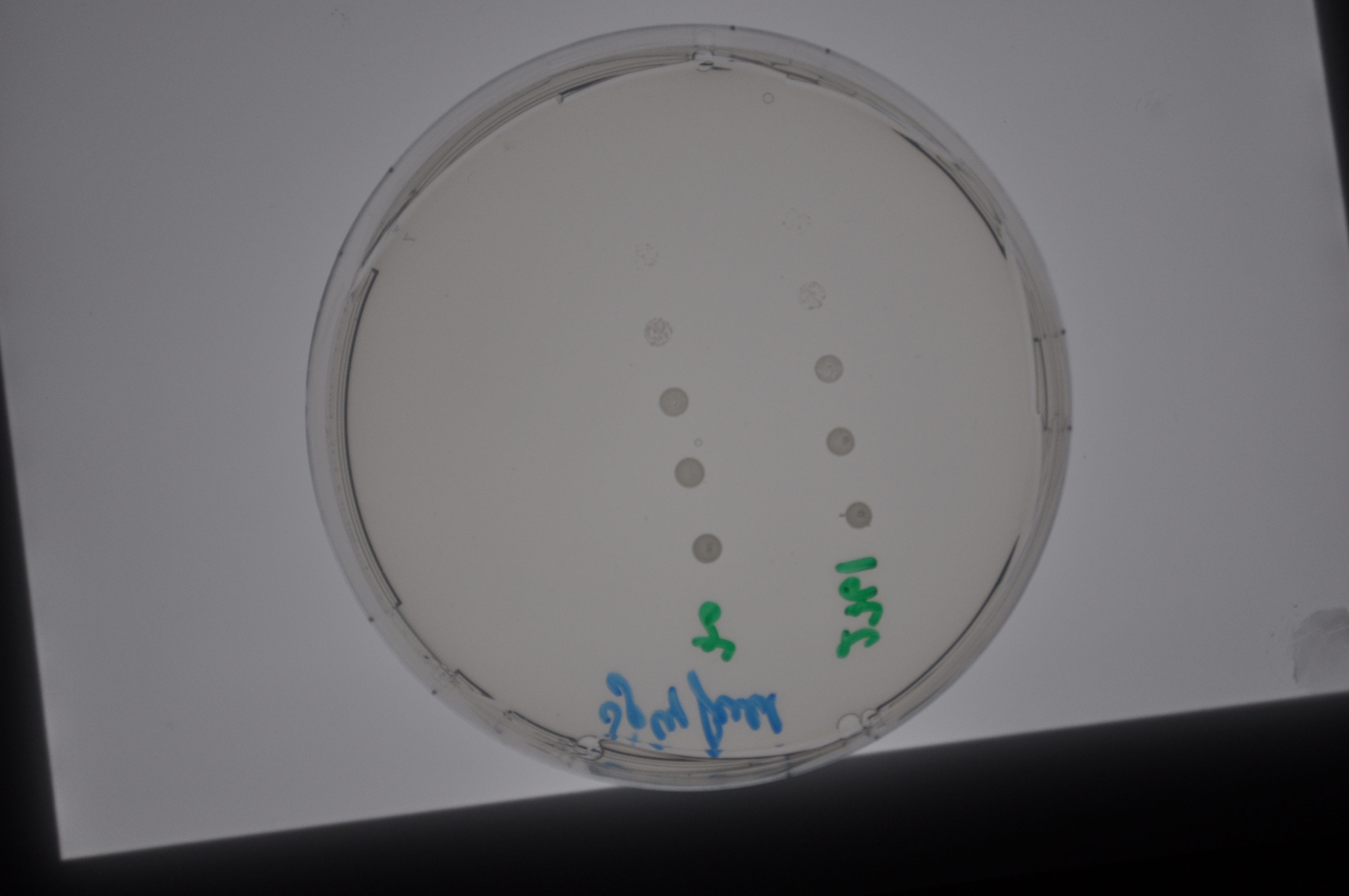

Supplement: Figure 4—figure supplement 3—source data 1. [file elife-69549-fig4-figsupp3-data1.zip › Figure4- figure supplement 3/Sglycerol_0atc.JPG]

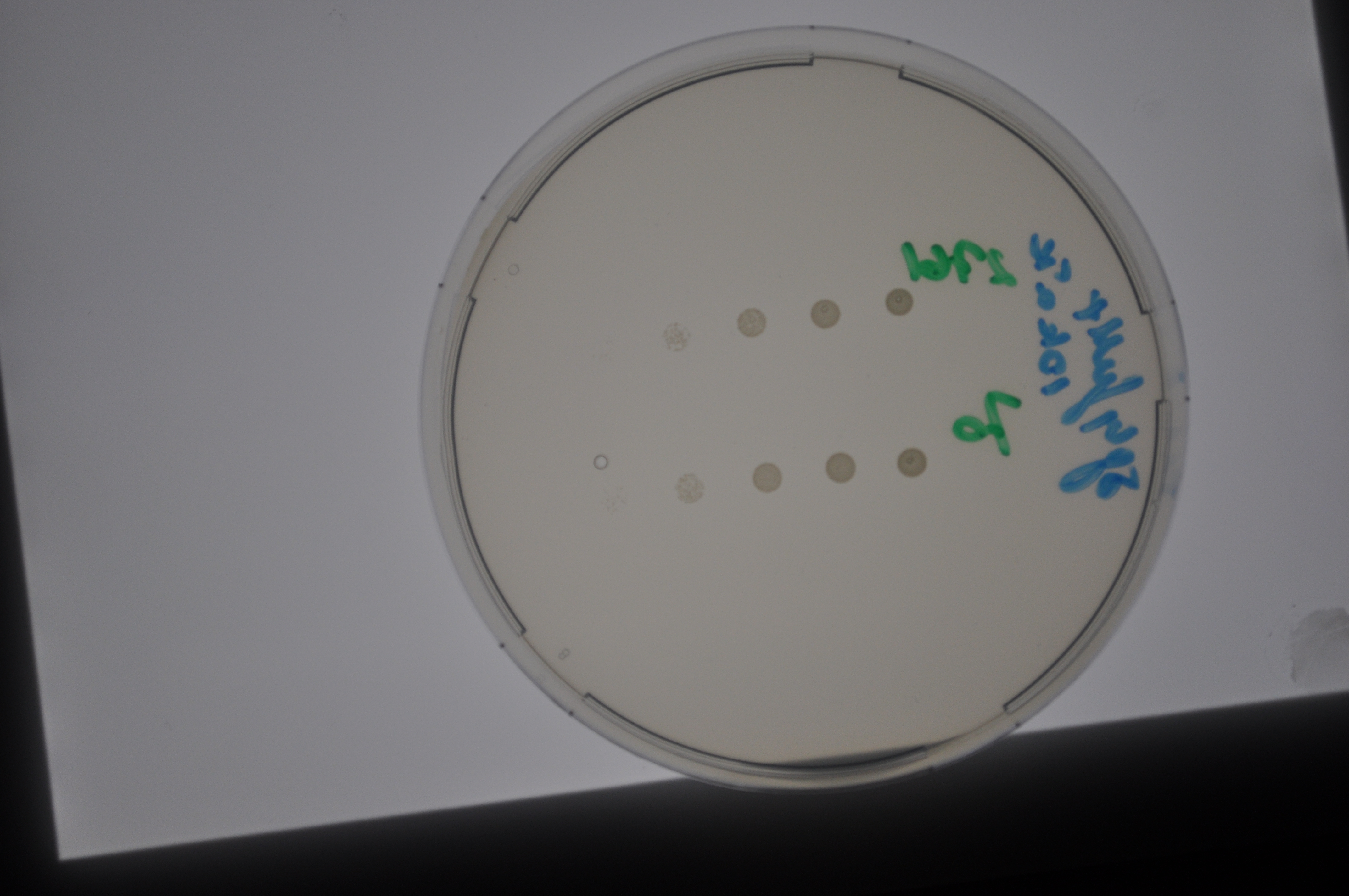

Supplement: Figure 4—figure supplement 3—source data 1. [file elife-69549-fig4-figsupp3-data1.zip › Figure4- figure supplement 3/Sglycerol_10katc.JPG]

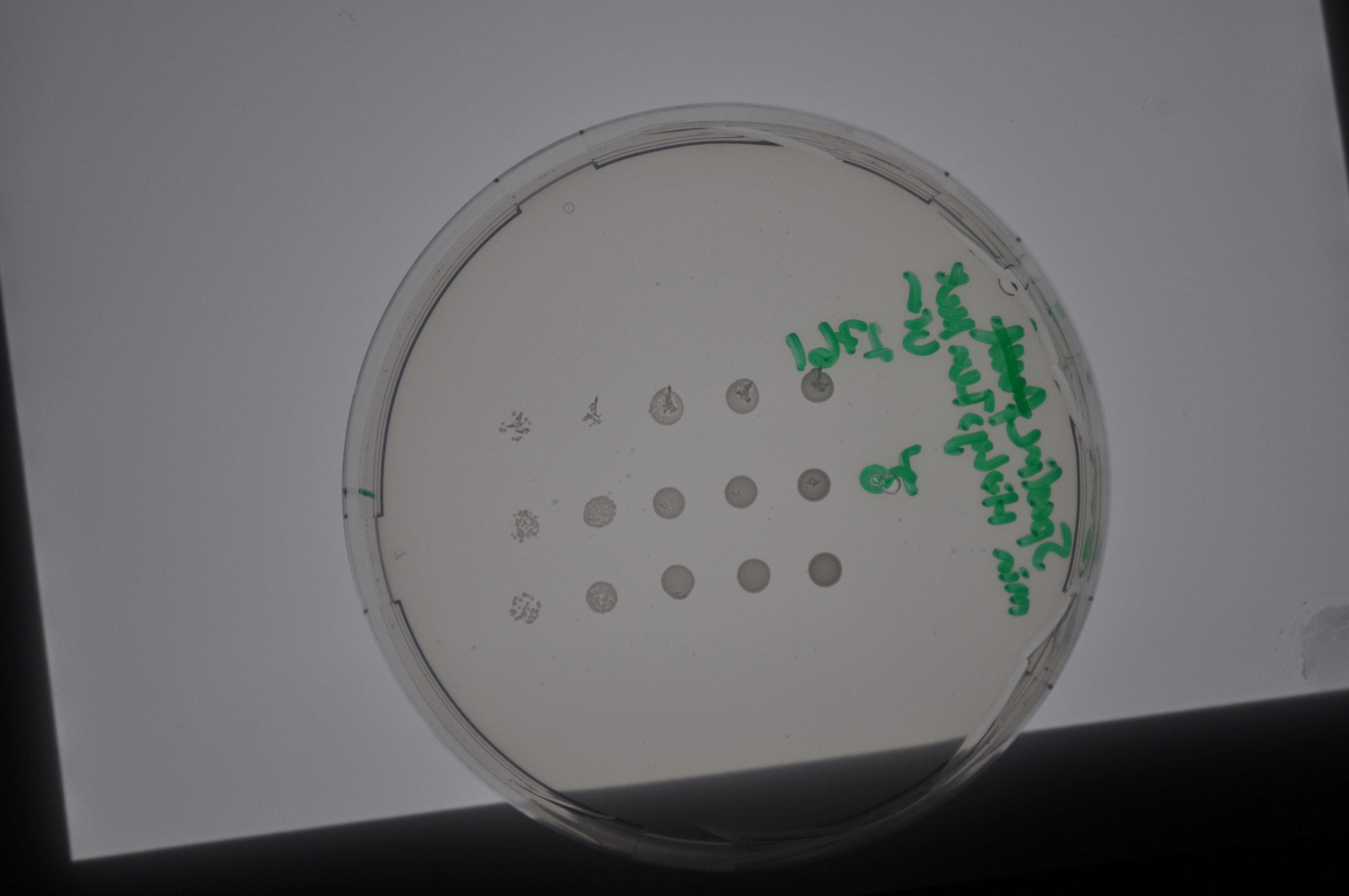

Supplement: Figure 4—figure supplement 3—source data 1. [file elife-69549-fig4-figsupp3-data1.zip › Figure4- figure supplement 3/Sproline_0aTc.JPG]

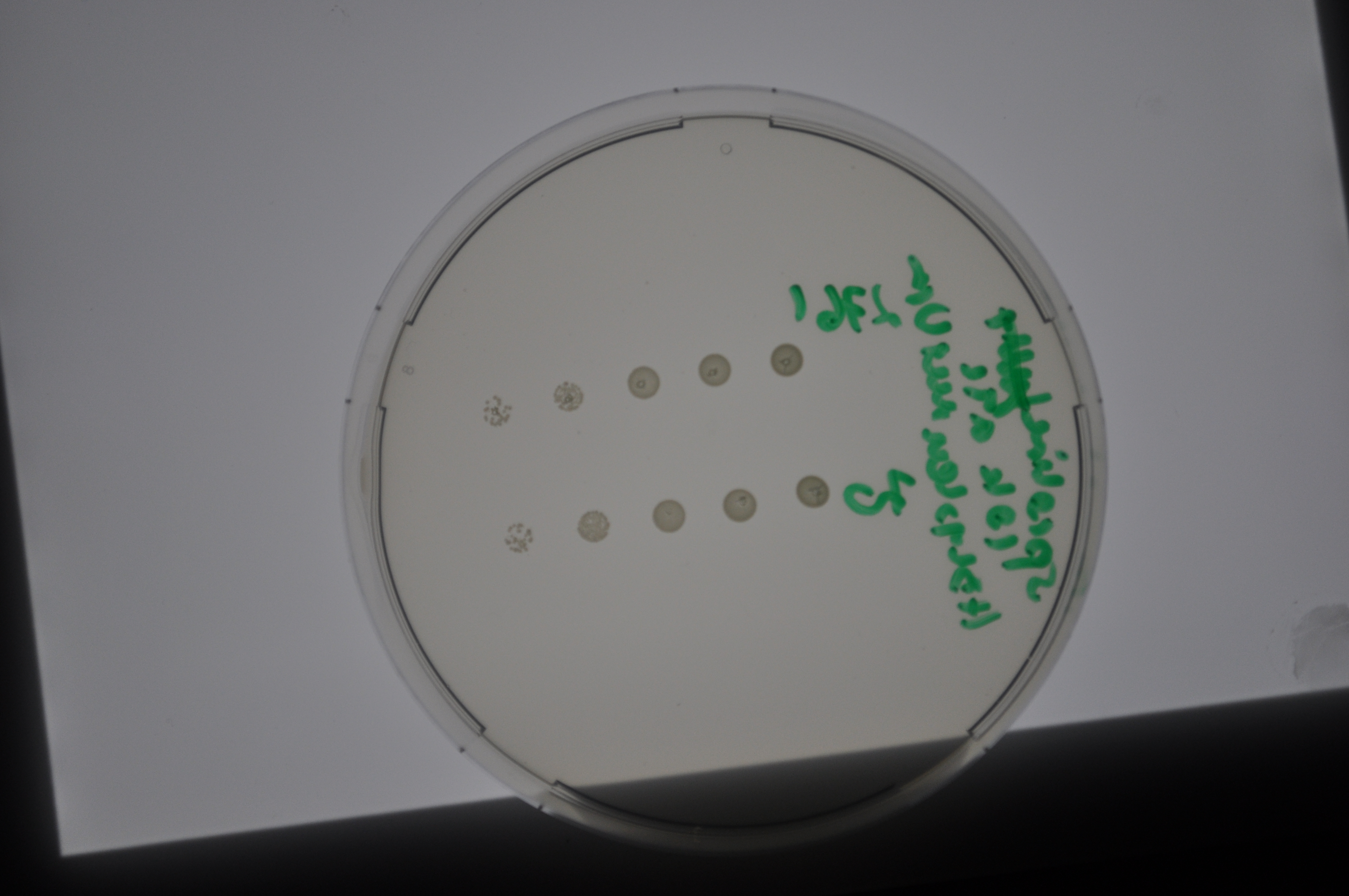

Supplement: Figure 4—figure supplement 3—source data 1. [file elife-69549-fig4-figsupp3-data1.zip › Figure4- figure supplement 3/SProline_10katc.JPG]

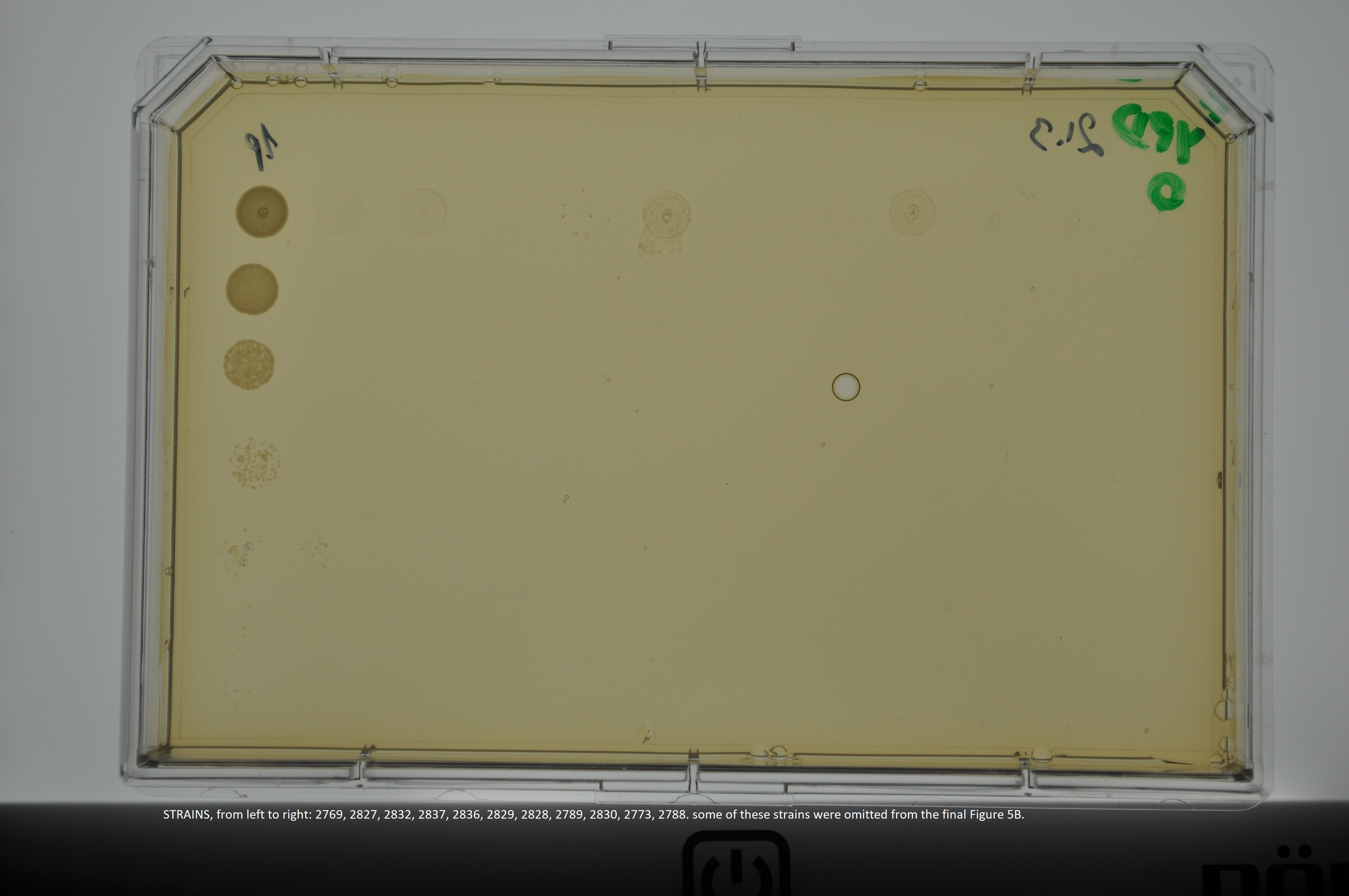

Supplement: Figure 5—source data 1. [file elife-69549-fig5-data1.zip › Figure 5/PanelB/YPD_0atc.JPG]

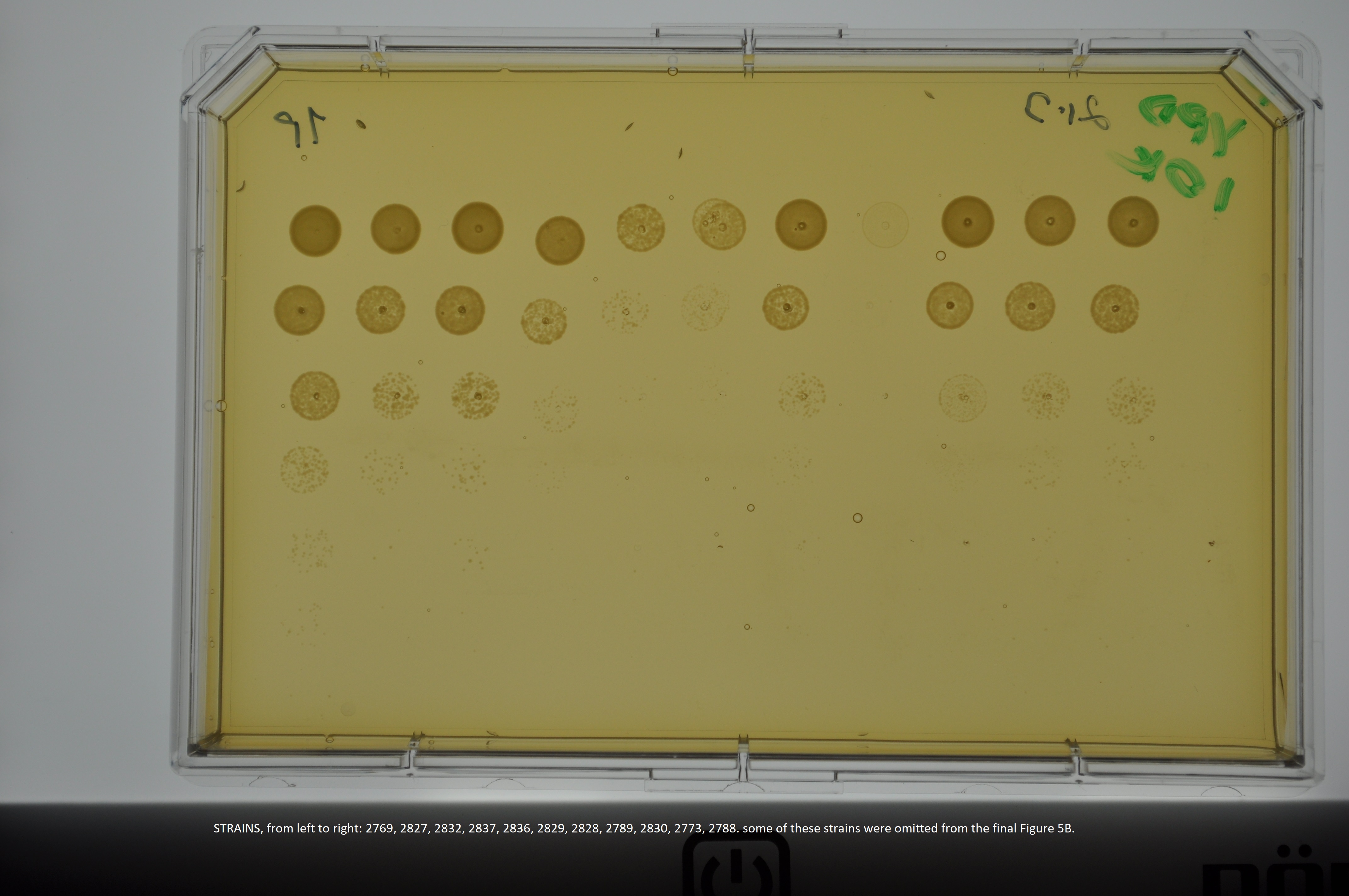

Supplement: Figure 5—source data 1. [file elife-69549-fig5-data1.zip › Figure 5/PanelB/YPD_10000atc.JPG]

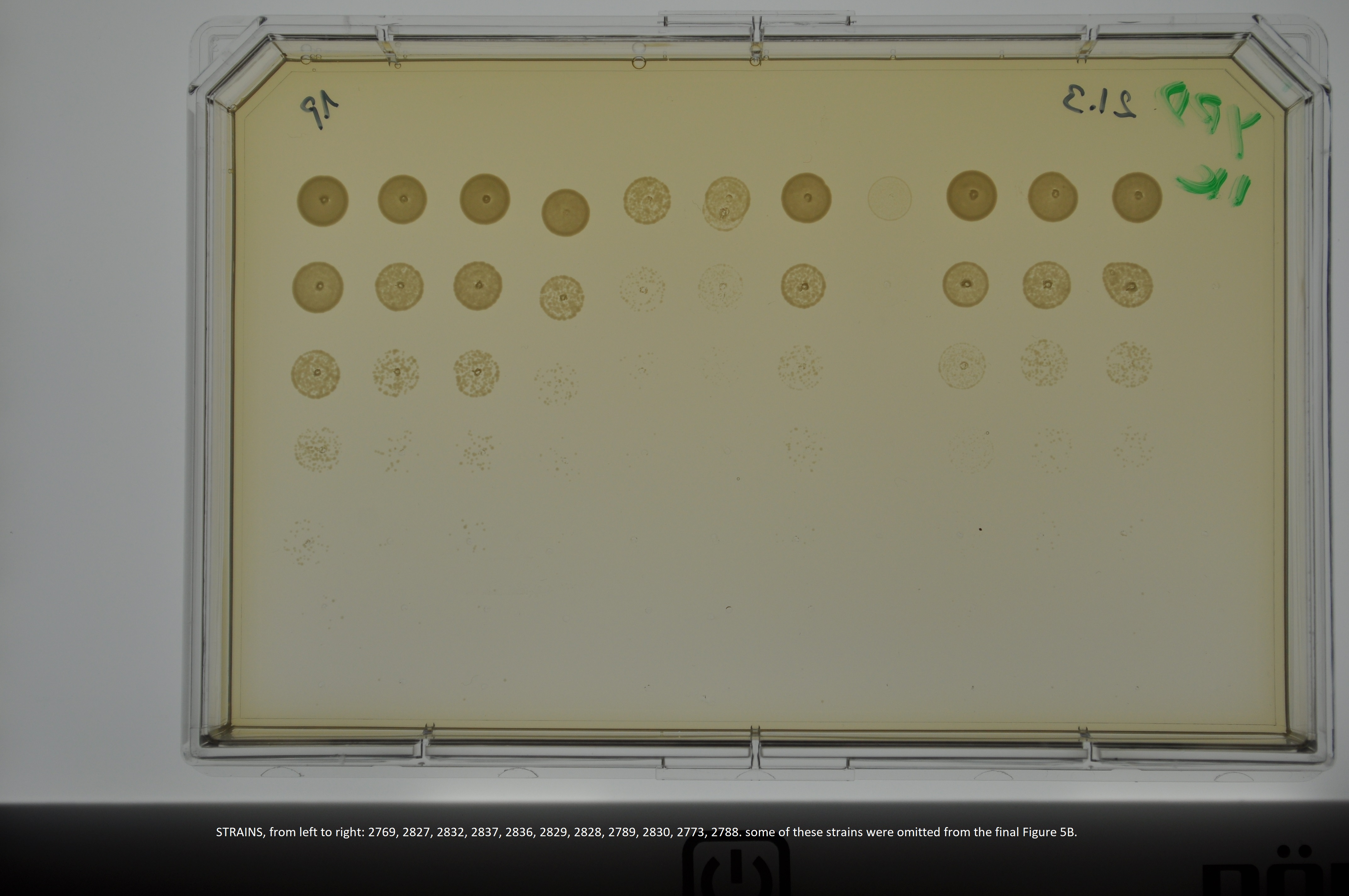

Supplement: Figure 5—source data 1. [file elife-69549-fig5-data1.zip › Figure 5/PanelB/YPD_1000atc.JPG]

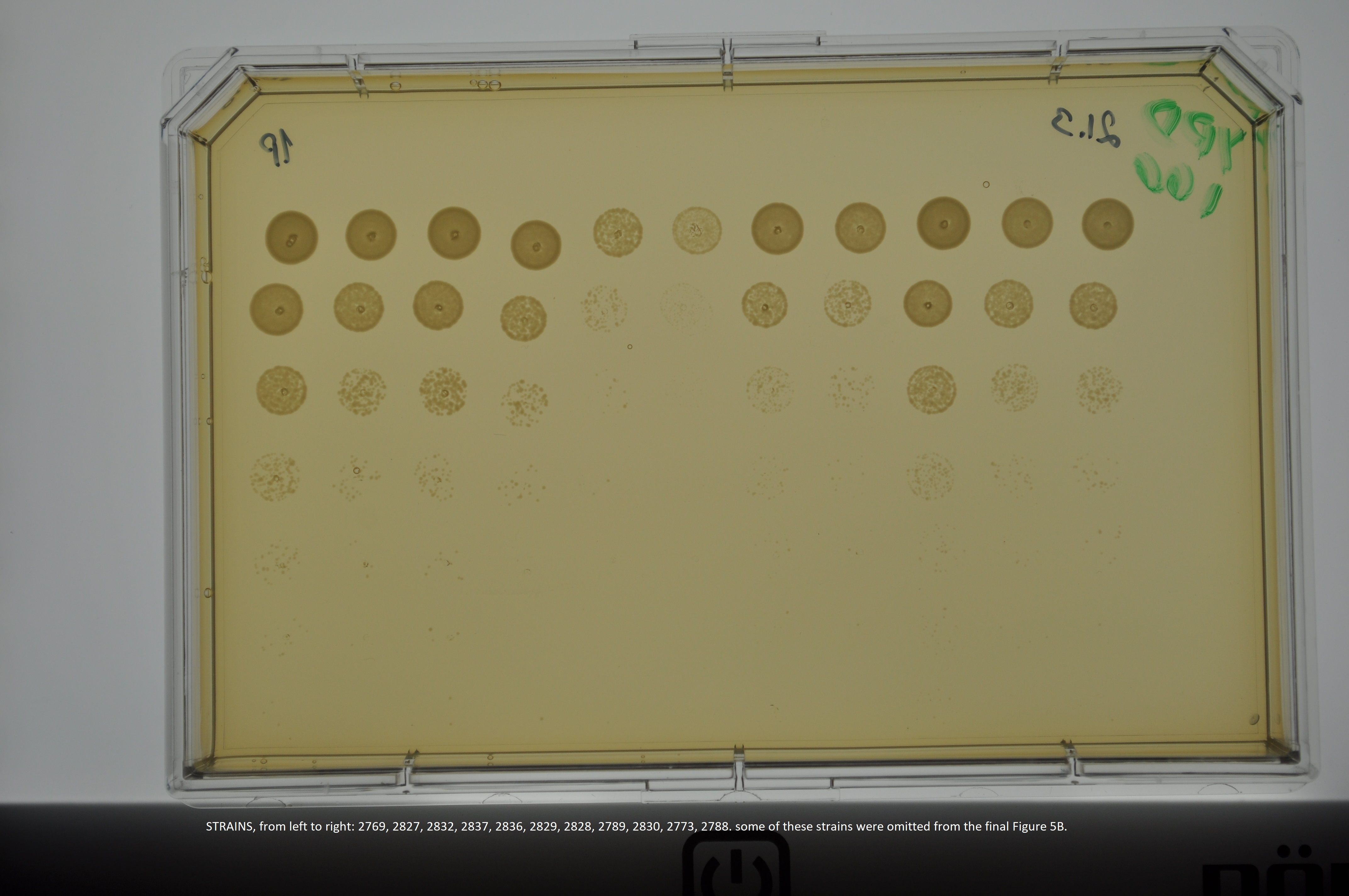

Supplement: Figure 5—source data 1. [file elife-69549-fig5-data1.zip › Figure 5/PanelB/YPD_100atc.JPG]

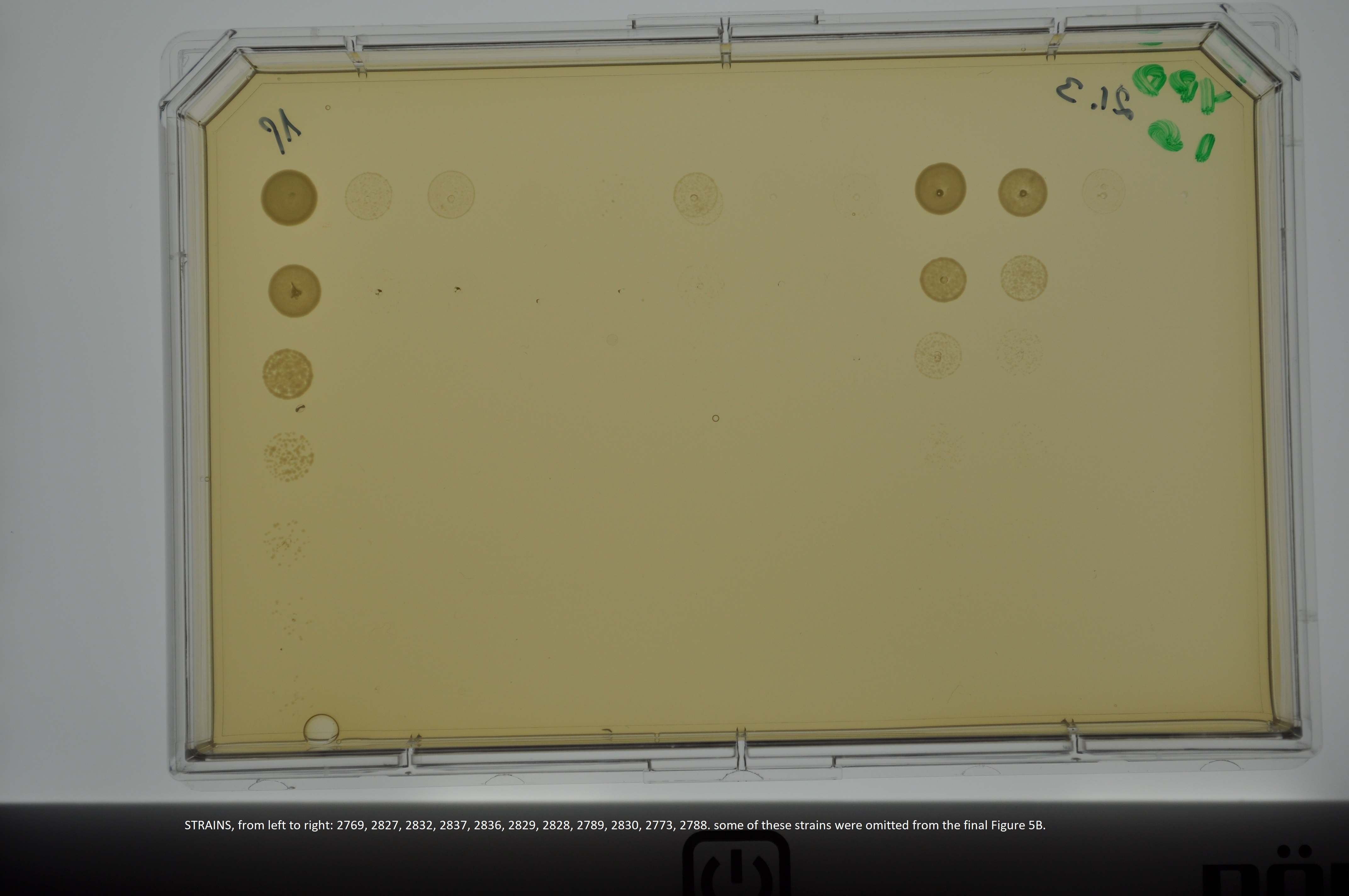

Supplement: Figure 5—source data 1. [file elife-69549-fig5-data1.zip › Figure 5/PanelB/YPD_10atc.JPG]

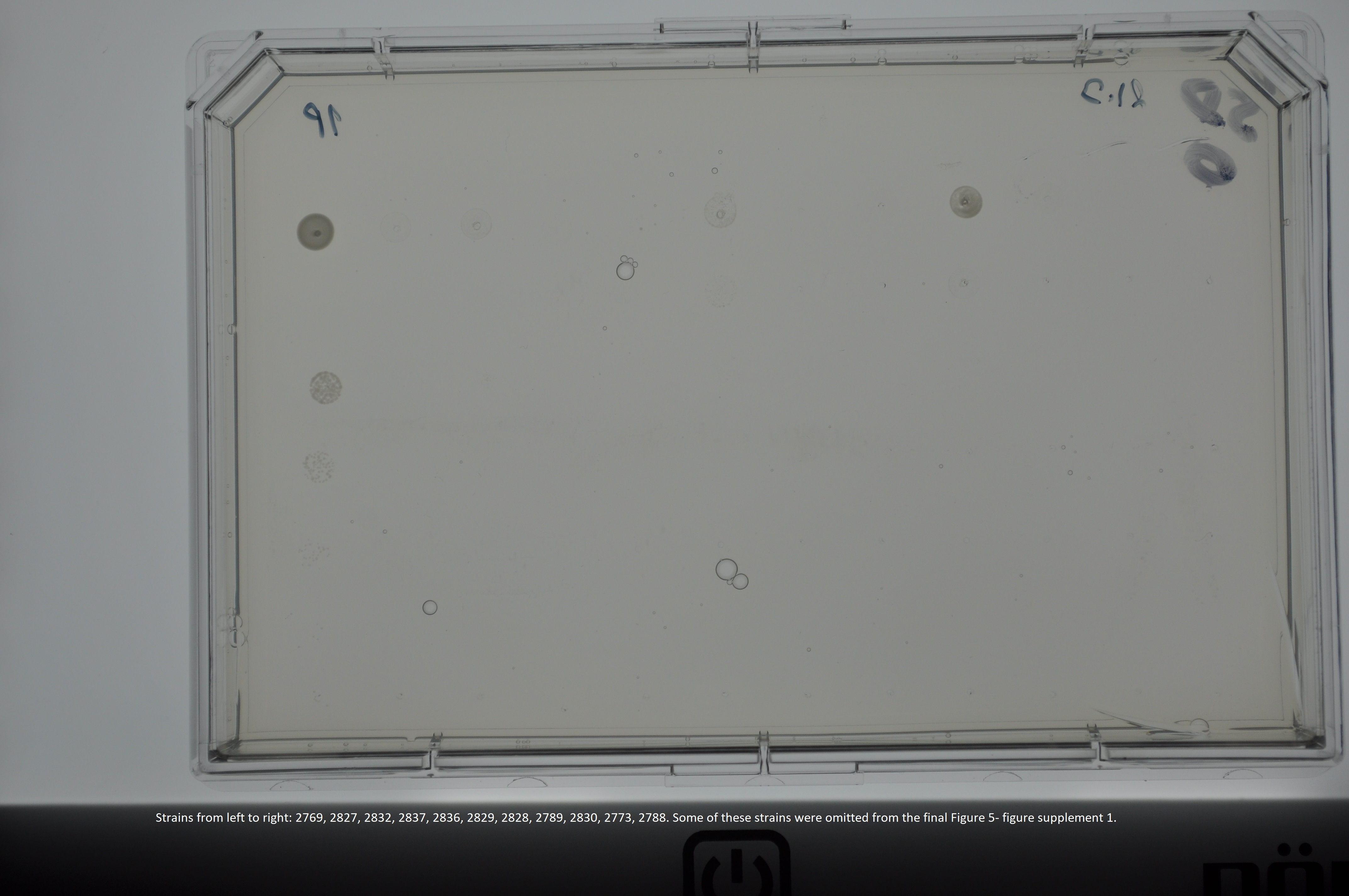

Supplement: Figure 5—figure supplement 1—source data 1. [file elife-69549-fig5-figsupp1-data1.zip › Figure5- figure supplement 1- source data 1/SD/SD_0atc.JPG]

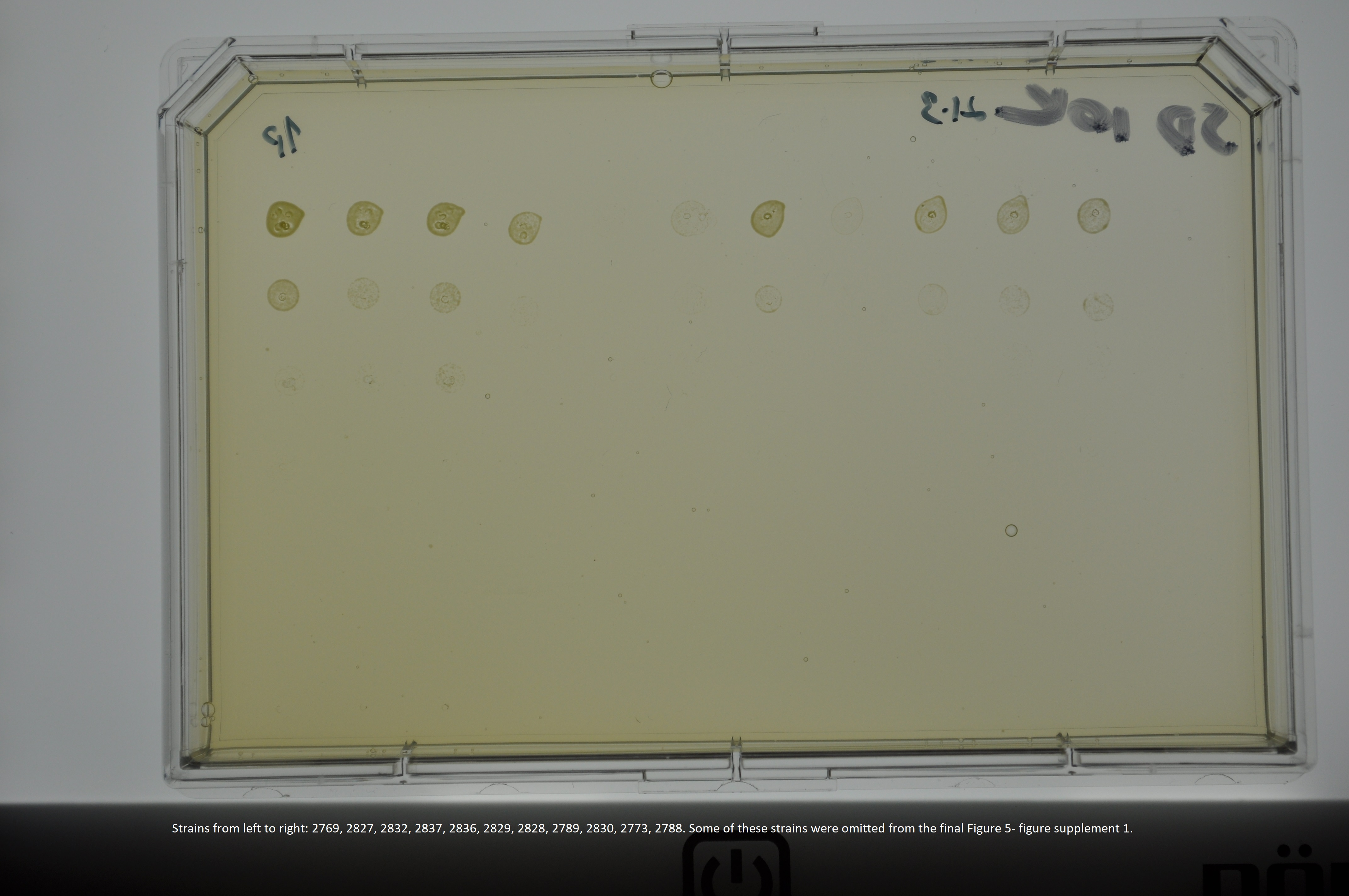

Supplement: Figure 5—figure supplement 1—source data 1. [file elife-69549-fig5-figsupp1-data1.zip › Figure5- figure supplement 1- source data 1/SD/SD_10000atc.JPG]

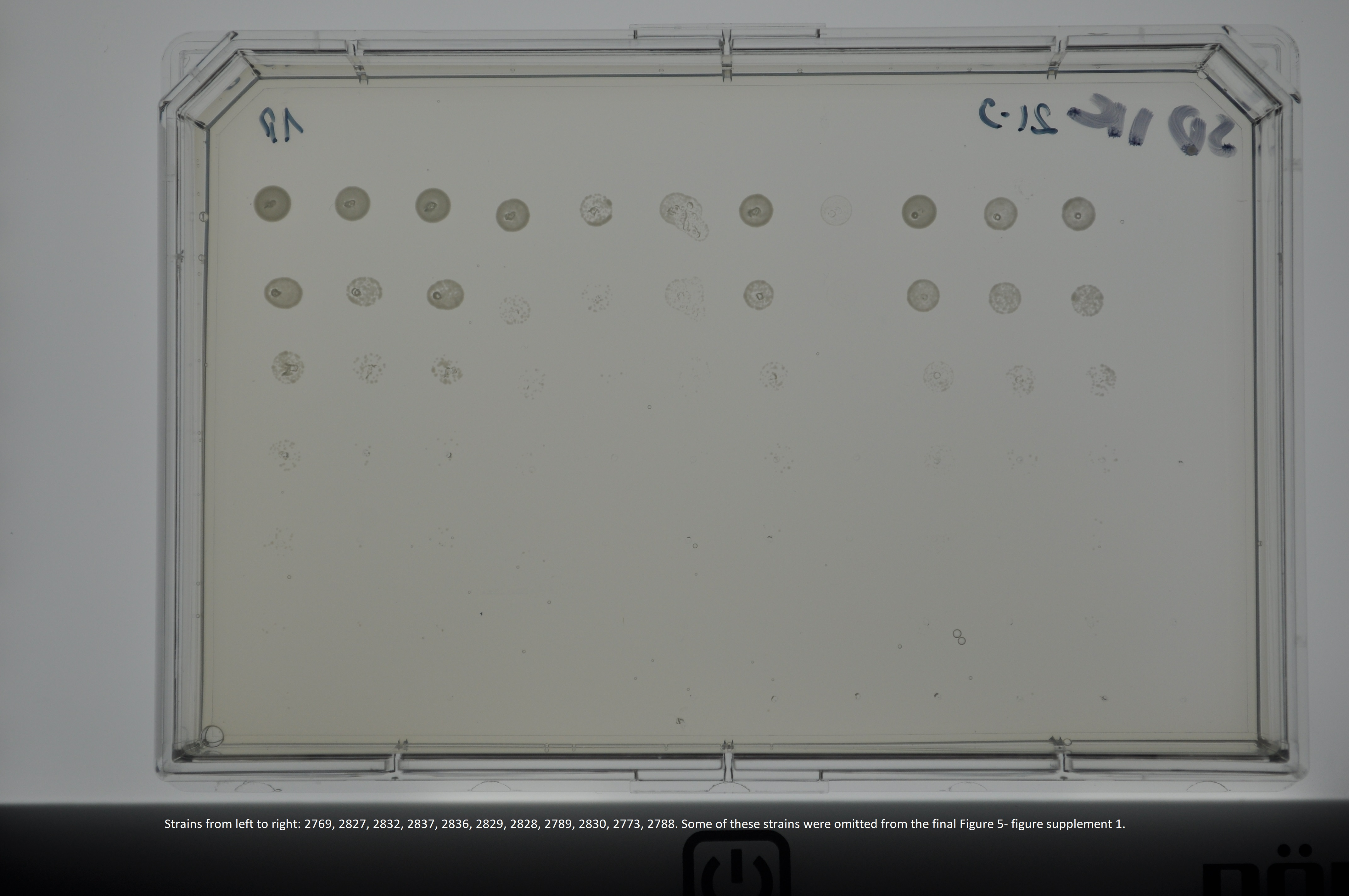

Supplement: Figure 5—figure supplement 1—source data 1. [file elife-69549-fig5-figsupp1-data1.zip › Figure5- figure supplement 1- source data 1/SD/SD_1000atc.JPG]

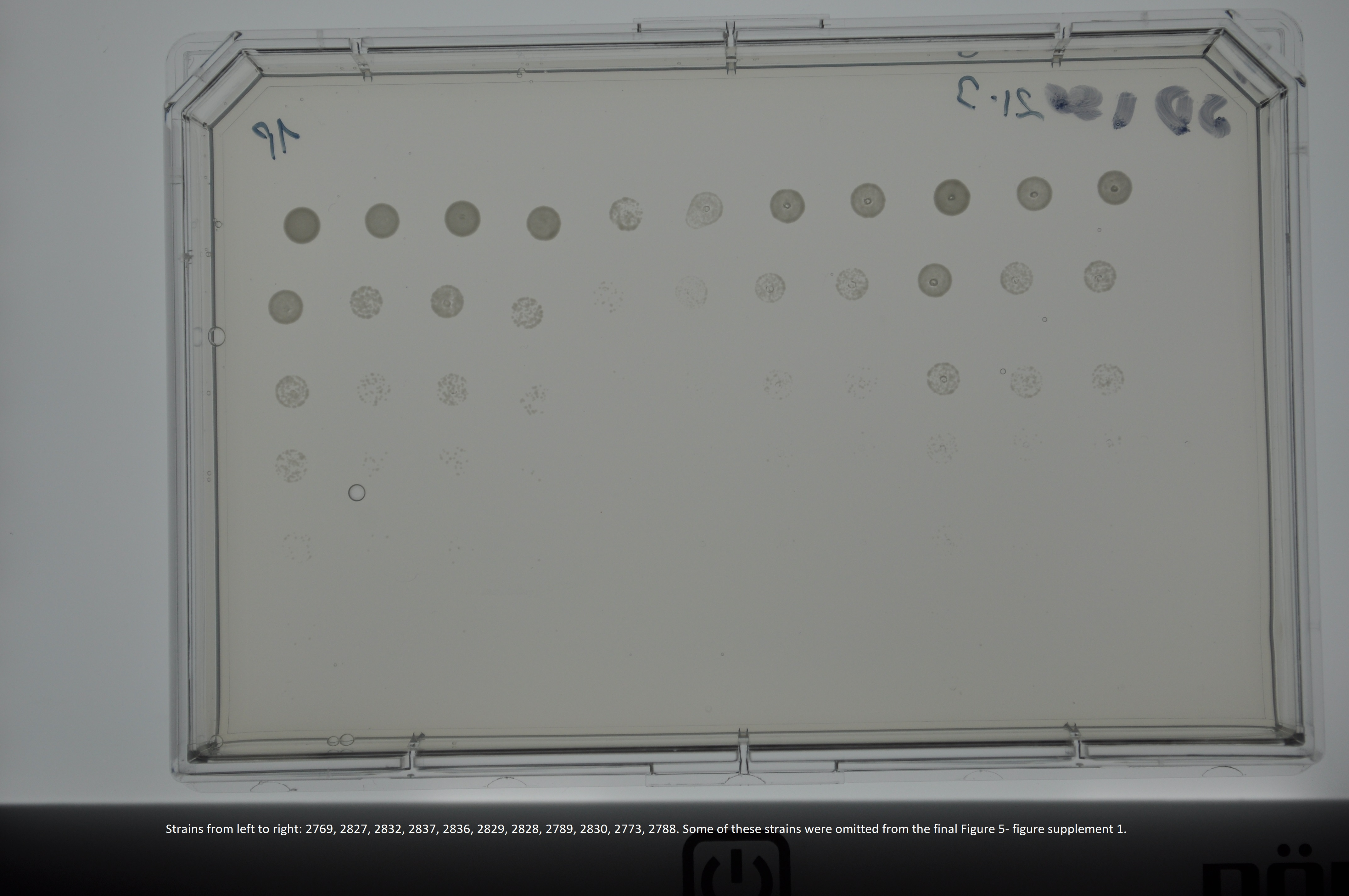

Supplement: Figure 5—figure supplement 1—source data 1. [file elife-69549-fig5-figsupp1-data1.zip › Figure5- figure supplement 1- source data 1/SD/SD_100atc.JPG]

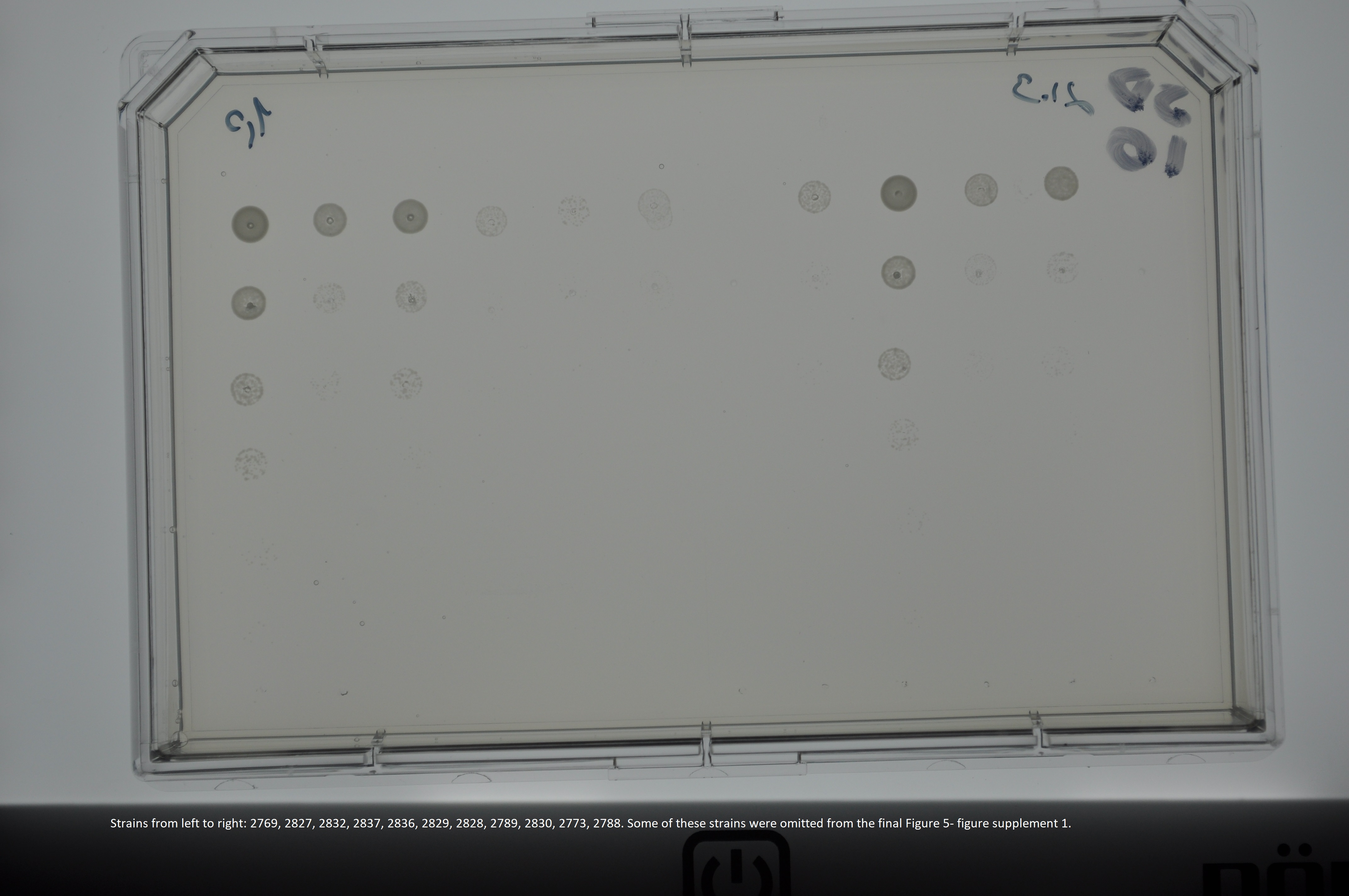

Supplement: Figure 5—figure supplement 1—source data 1. [file elife-69549-fig5-figsupp1-data1.zip › Figure5- figure supplement 1- source data 1/SD/SD_10atc.JPG]

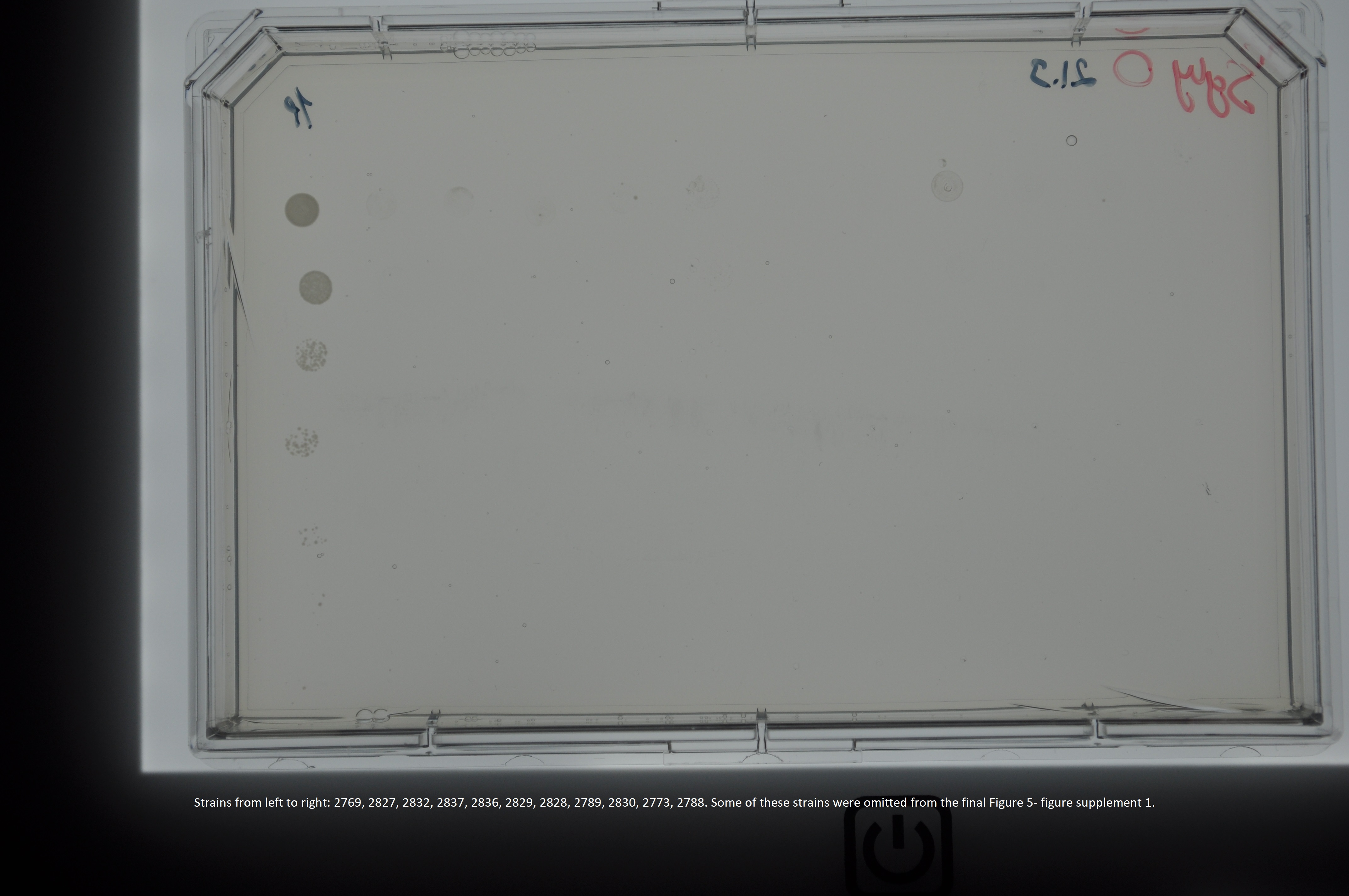

Supplement: Figure 5—figure supplement 1—source data 1. [file elife-69549-fig5-figsupp1-data1.zip › Figure5- figure supplement 1- source data 1/Sglycerol/Sglycerol_0atc.JPG]

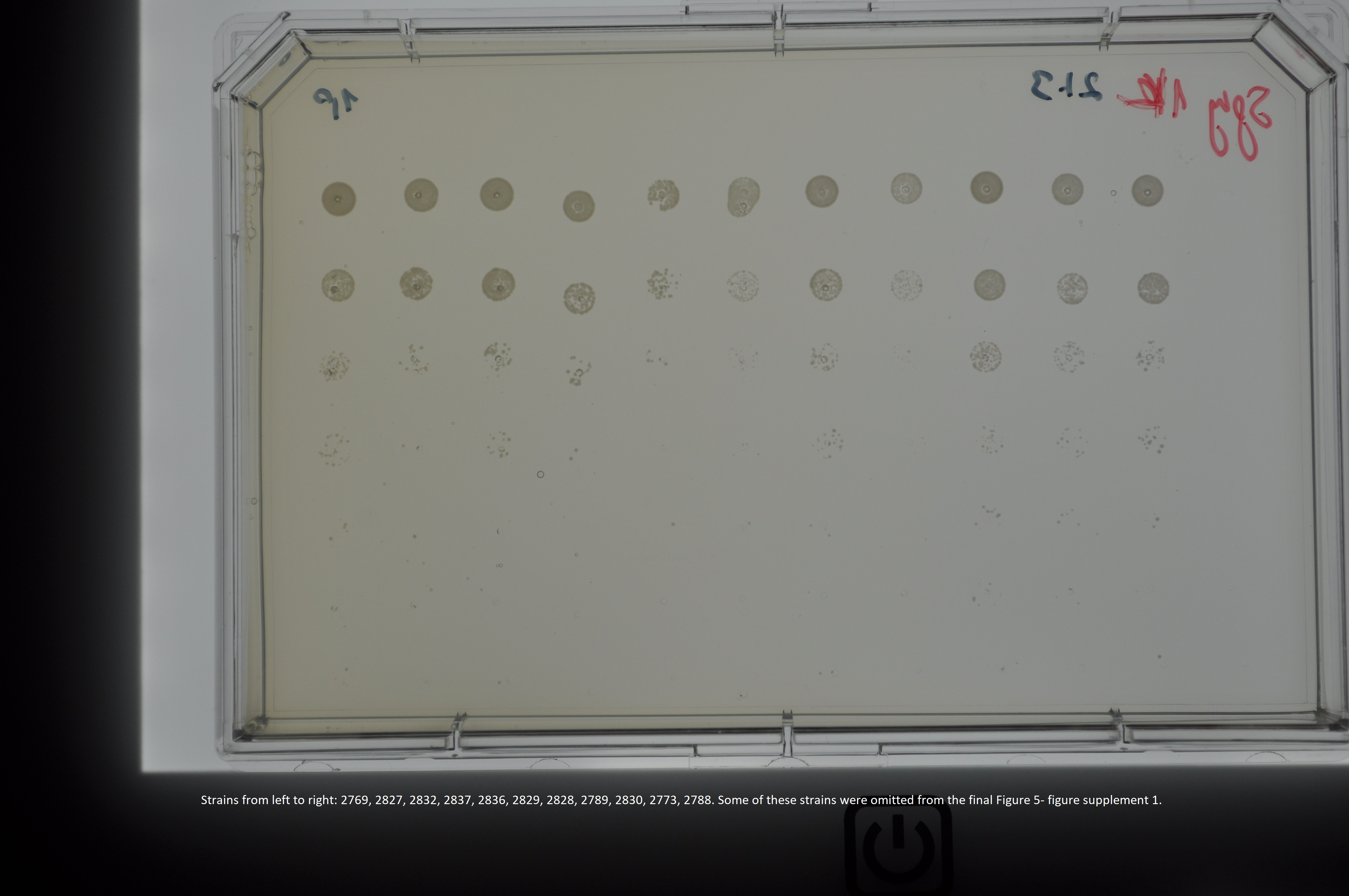

Supplement: Figure 5—figure supplement 1—source data 1. [file elife-69549-fig5-figsupp1-data1.zip › Figure5- figure supplement 1- source data 1/Sglycerol/Sglycerol_1000atc.JPG]

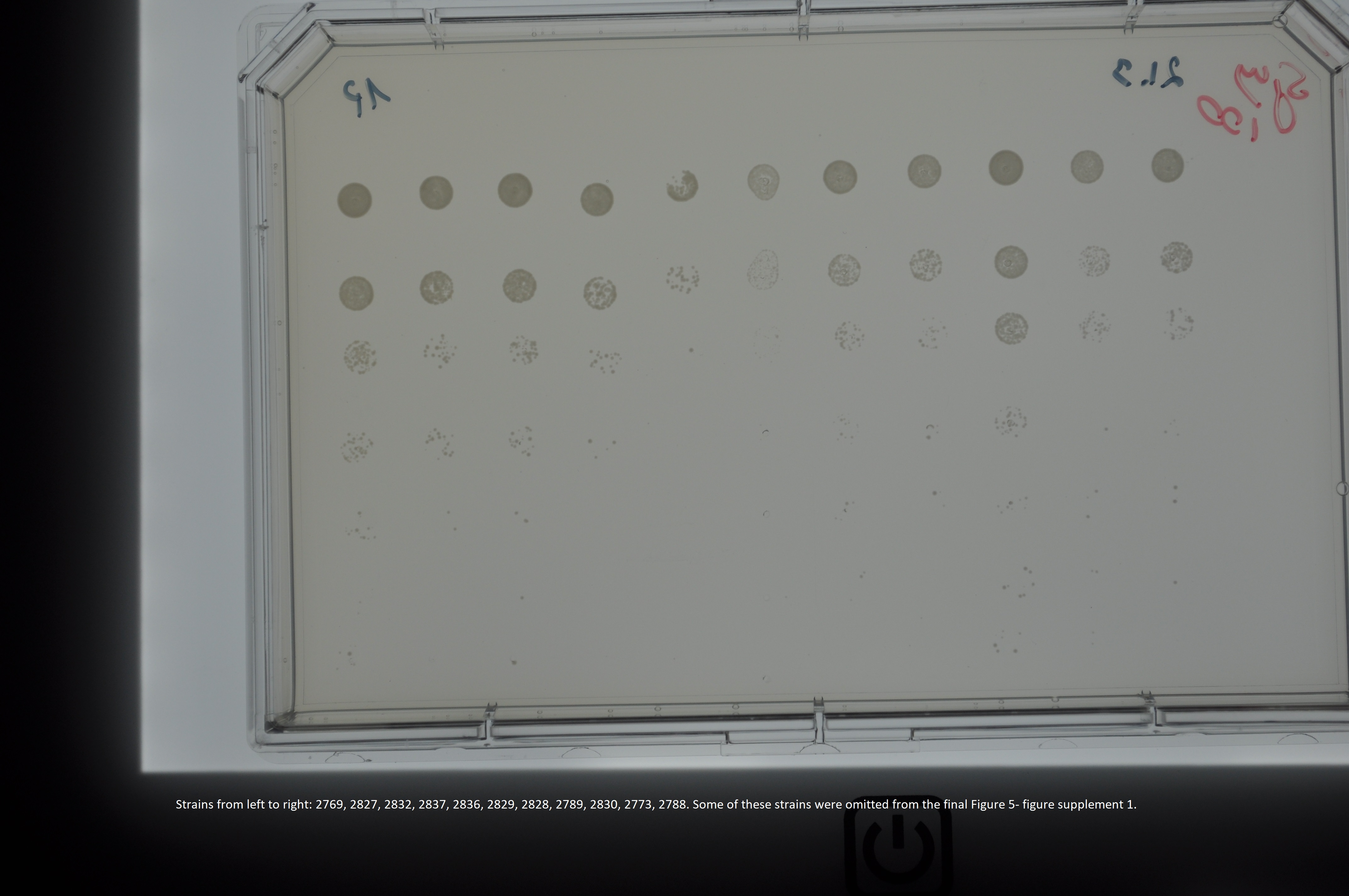

Supplement: Figure 5—figure supplement 1—source data 1. [file elife-69549-fig5-figsupp1-data1.zip › Figure5- figure supplement 1- source data 1/Sglycerol/Sglycerol_100atc.JPG]

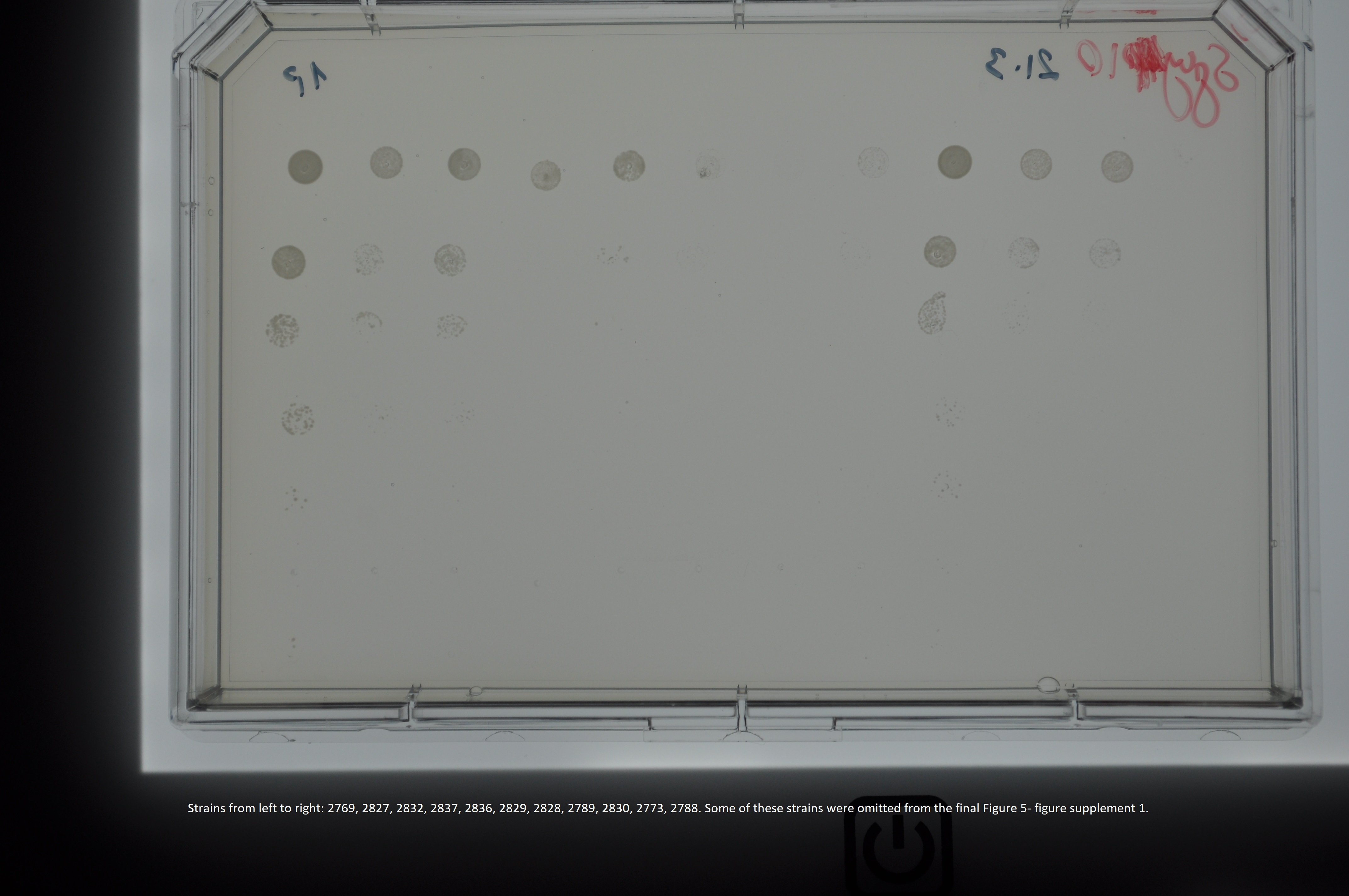

Supplement: Figure 5—figure supplement 1—source data 1. [file elife-69549-fig5-figsupp1-data1.zip › Figure5- figure supplement 1- source data 1/Sglycerol/Sglycerol_10atc.JPG]

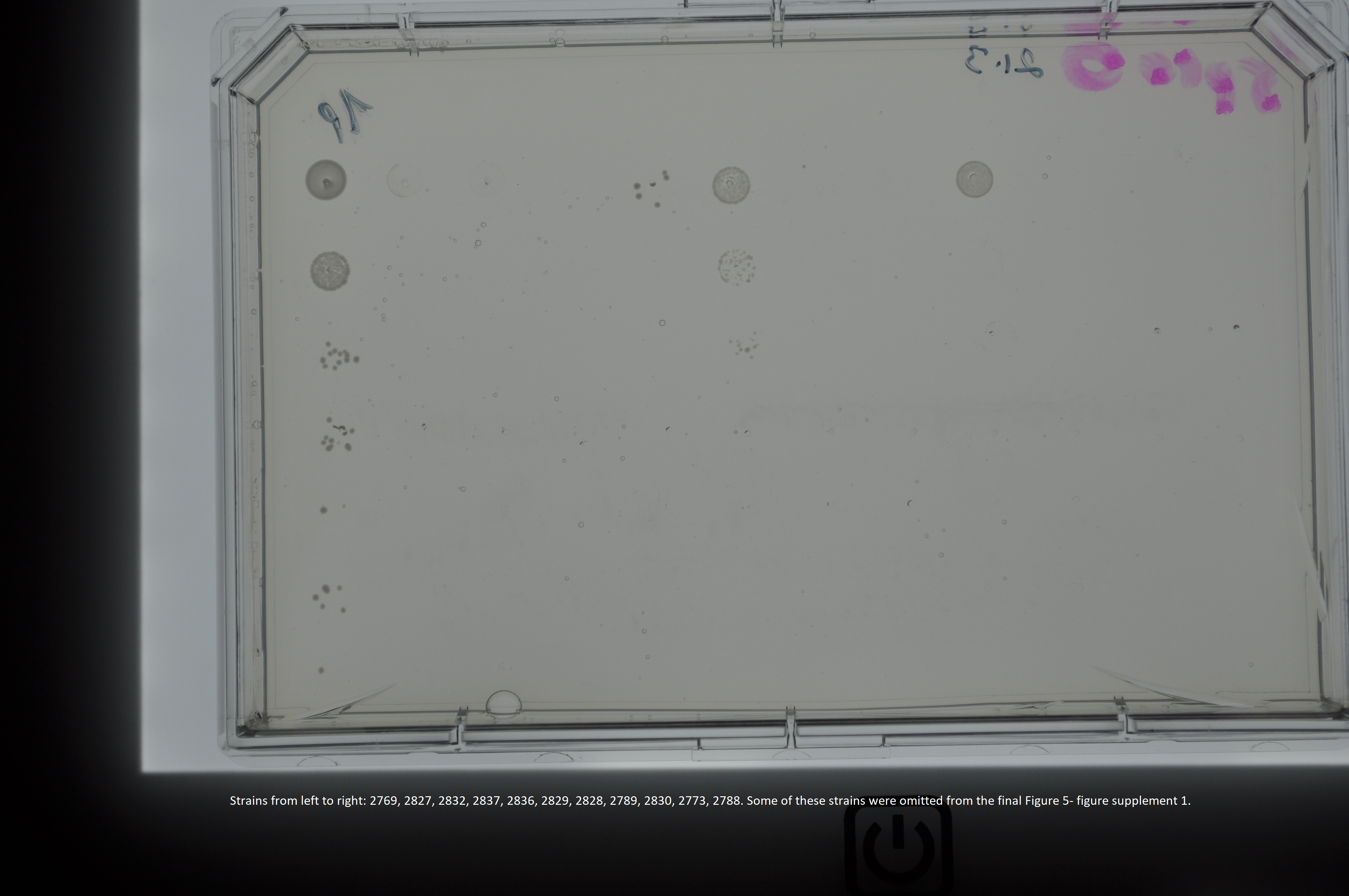

Supplement: Figure 5—figure supplement 1—source data 1. [file elife-69549-fig5-figsupp1-data1.zip › Figure5- figure supplement 1- source data 1/SProline/SPro_0atc.JPG]

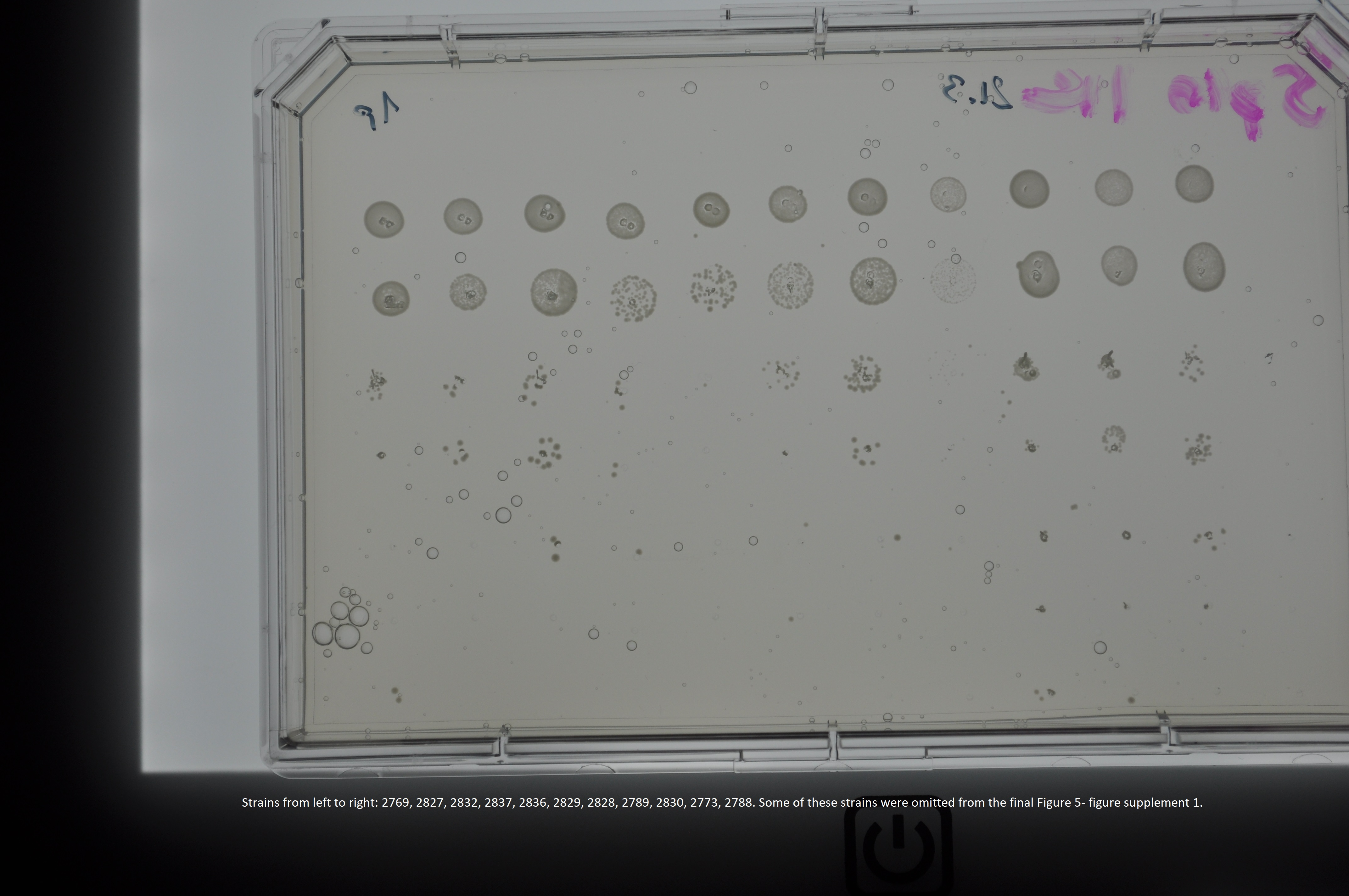

Supplement: Figure 5—figure supplement 1—source data 1. [file elife-69549-fig5-figsupp1-data1.zip › Figure5- figure supplement 1- source data 1/SProline/SPro_1000atc.JPG]

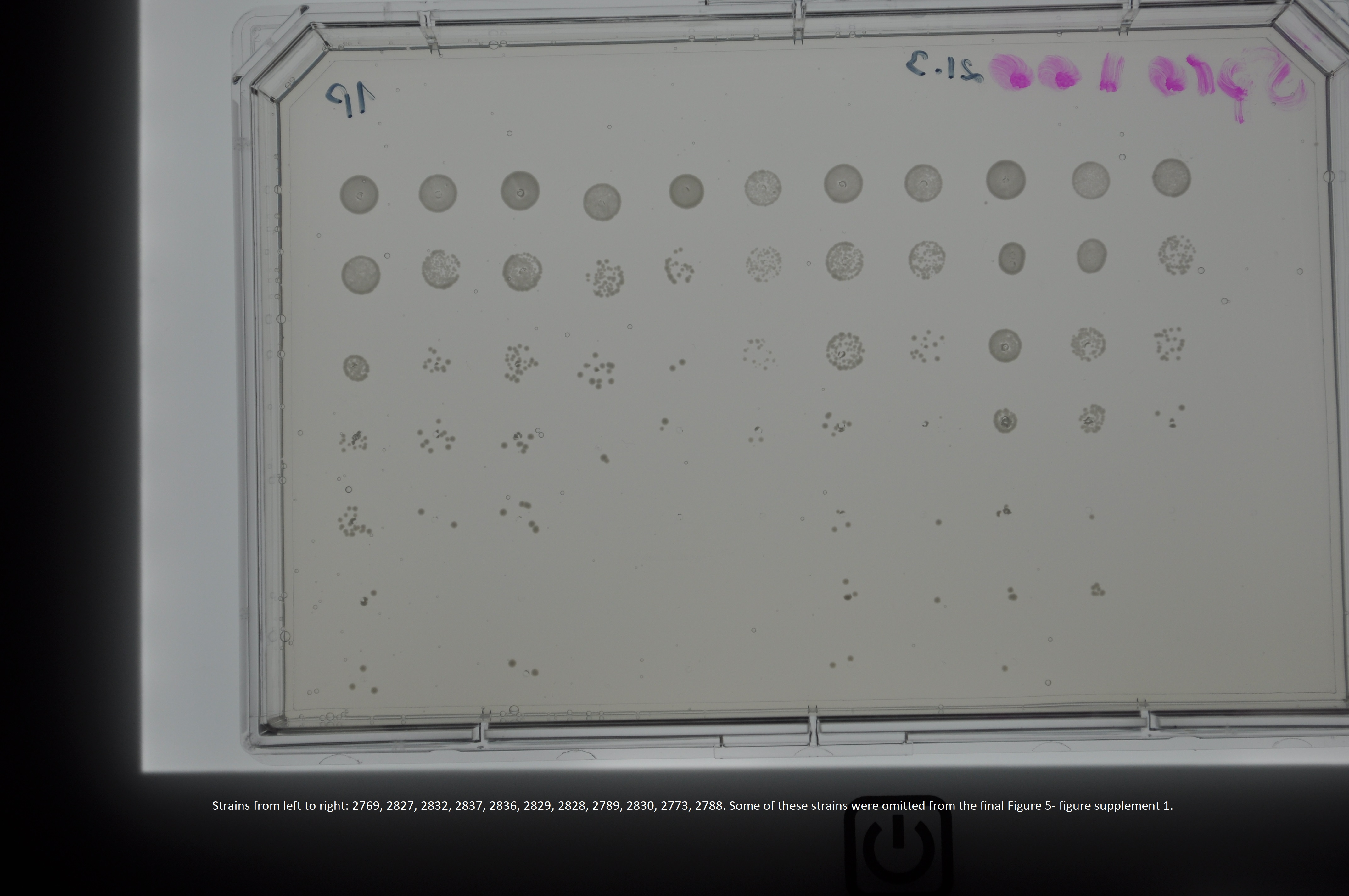

Supplement: Figure 5—figure supplement 1—source data 1. [file elife-69549-fig5-figsupp1-data1.zip › Figure5- figure supplement 1- source data 1/SProline/SPro_100atc.JPG]

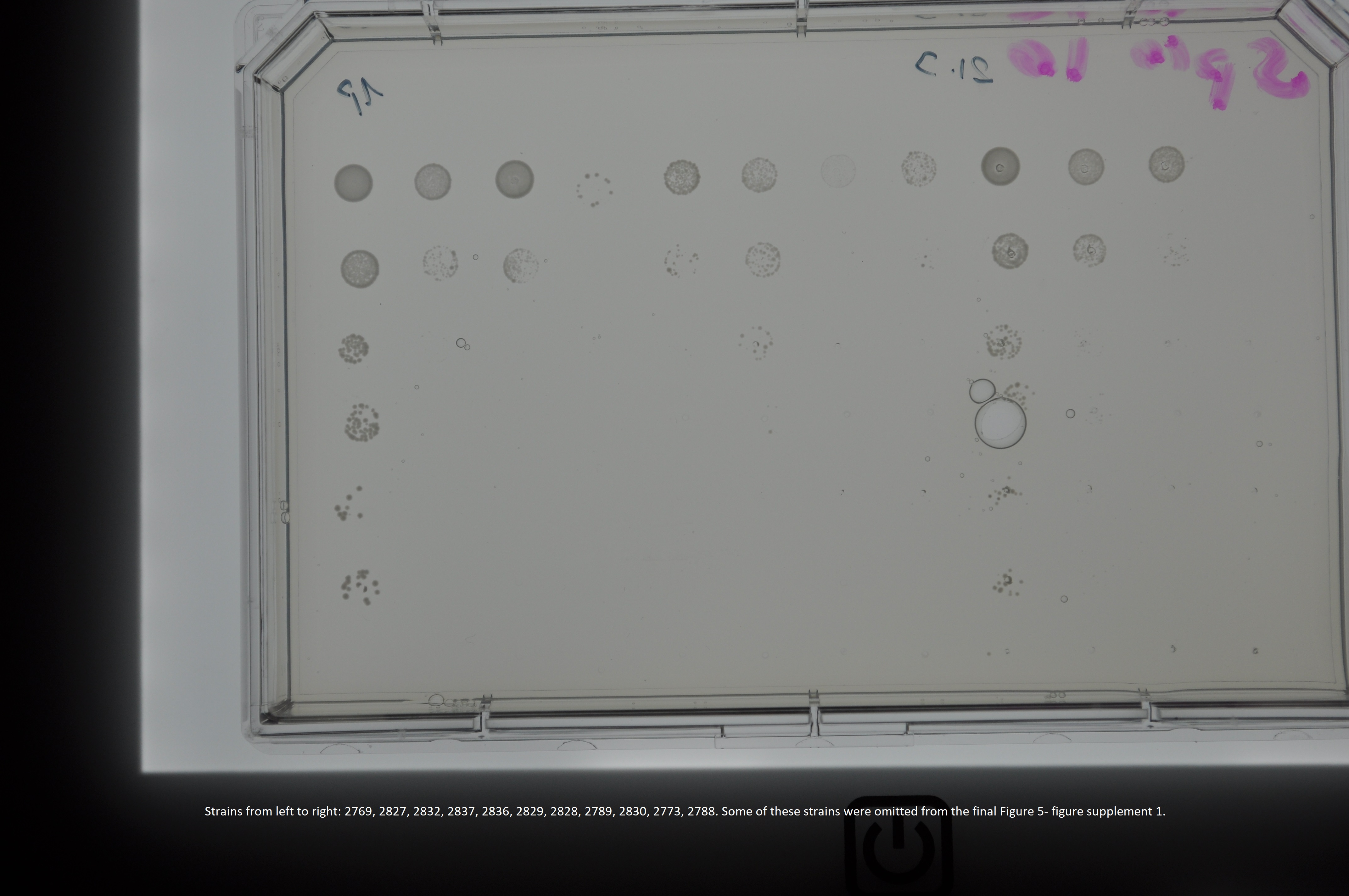

Supplement: Figure 5—figure supplement 1—source data 1. [file elife-69549-fig5-figsupp1-data1.zip › Figure5- figure supplement 1- source data 1/SProline/SPro_10atc.JPG]

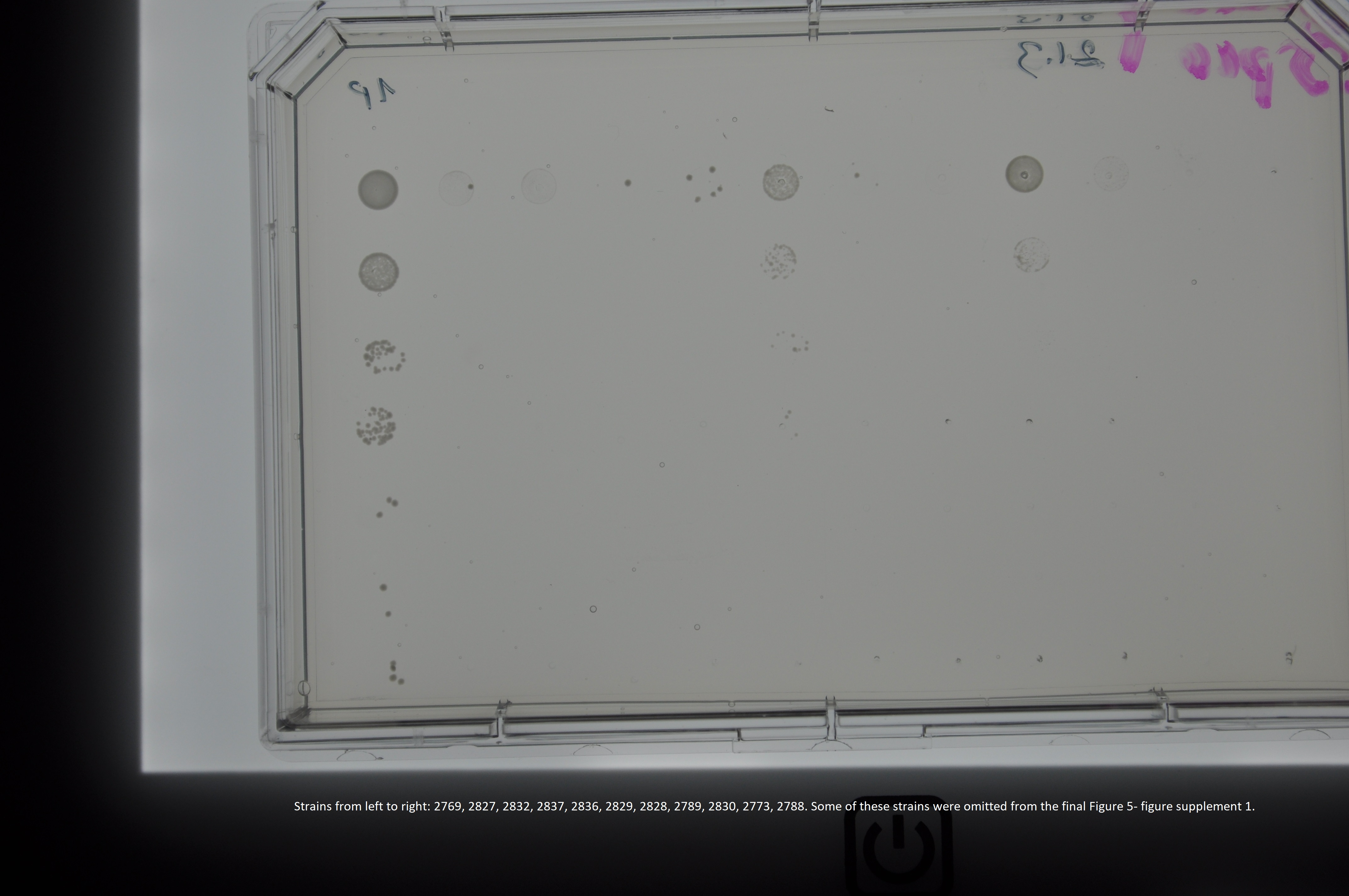

Supplement: Figure 5—figure supplement 1—source data 1. [file elife-69549-fig5-figsupp1-data1.zip › Figure5- figure supplement 1- source data 1/SProline/SPro_1atc.JPG]

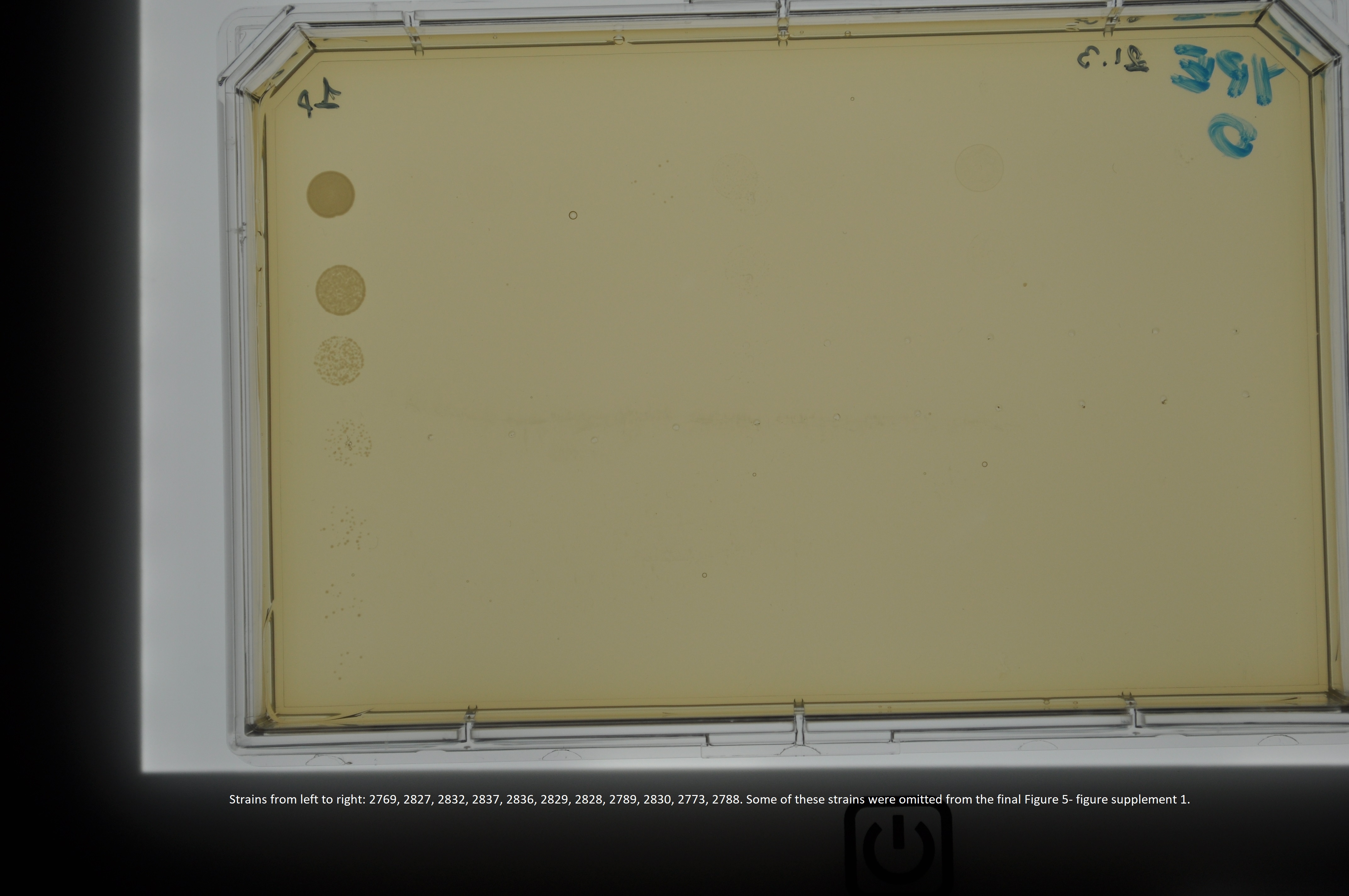

Supplement: Figure 5—figure supplement 1—source data 1. [file elife-69549-fig5-figsupp1-data1.zip › Figure5- figure supplement 1- source data 1/YPE/YPE_0atc.JPG]

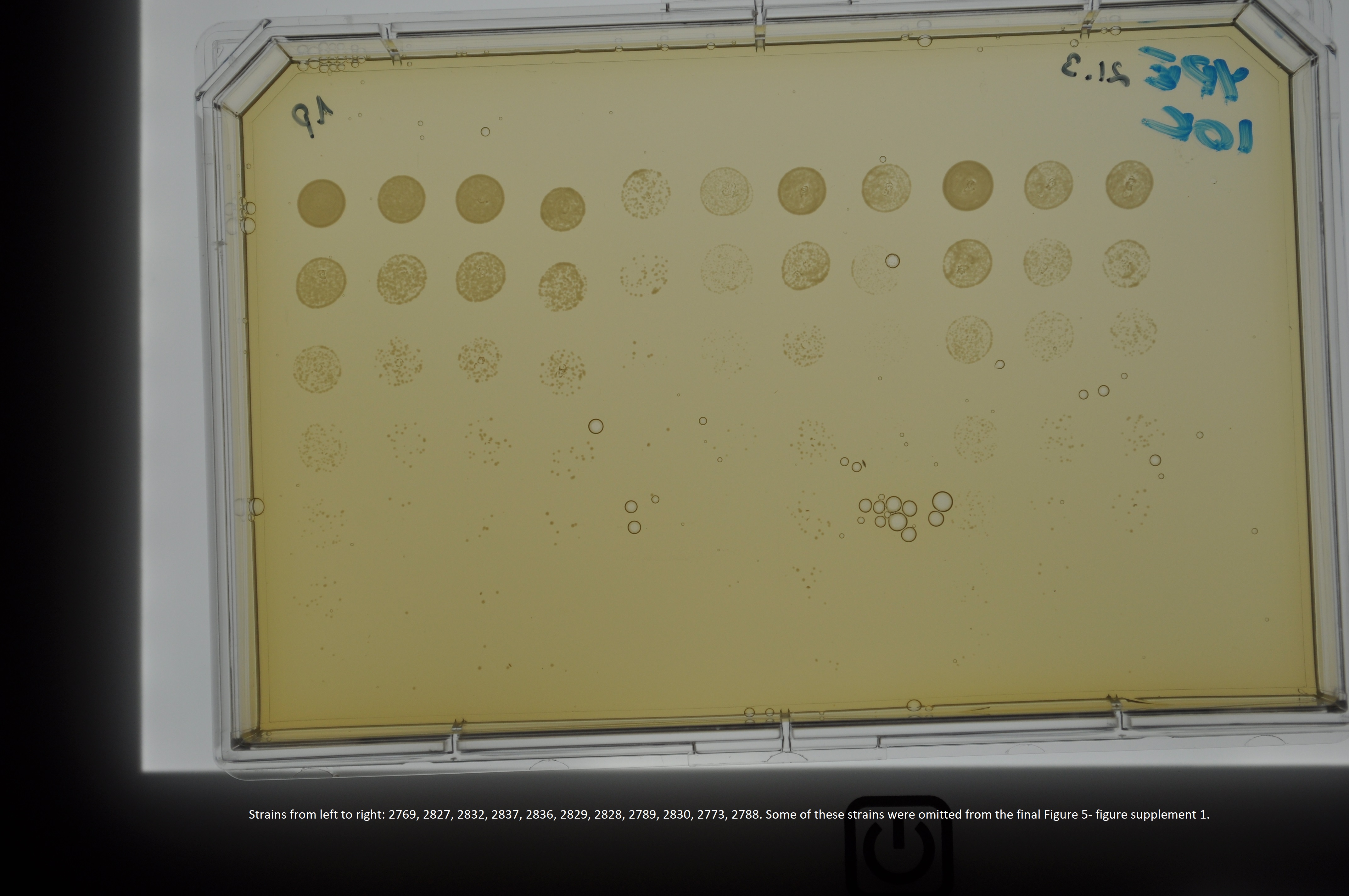

Supplement: Figure 5—figure supplement 1—source data 1. [file elife-69549-fig5-figsupp1-data1.zip › Figure5- figure supplement 1- source data 1/YPE/YPE_10000atc.JPG]

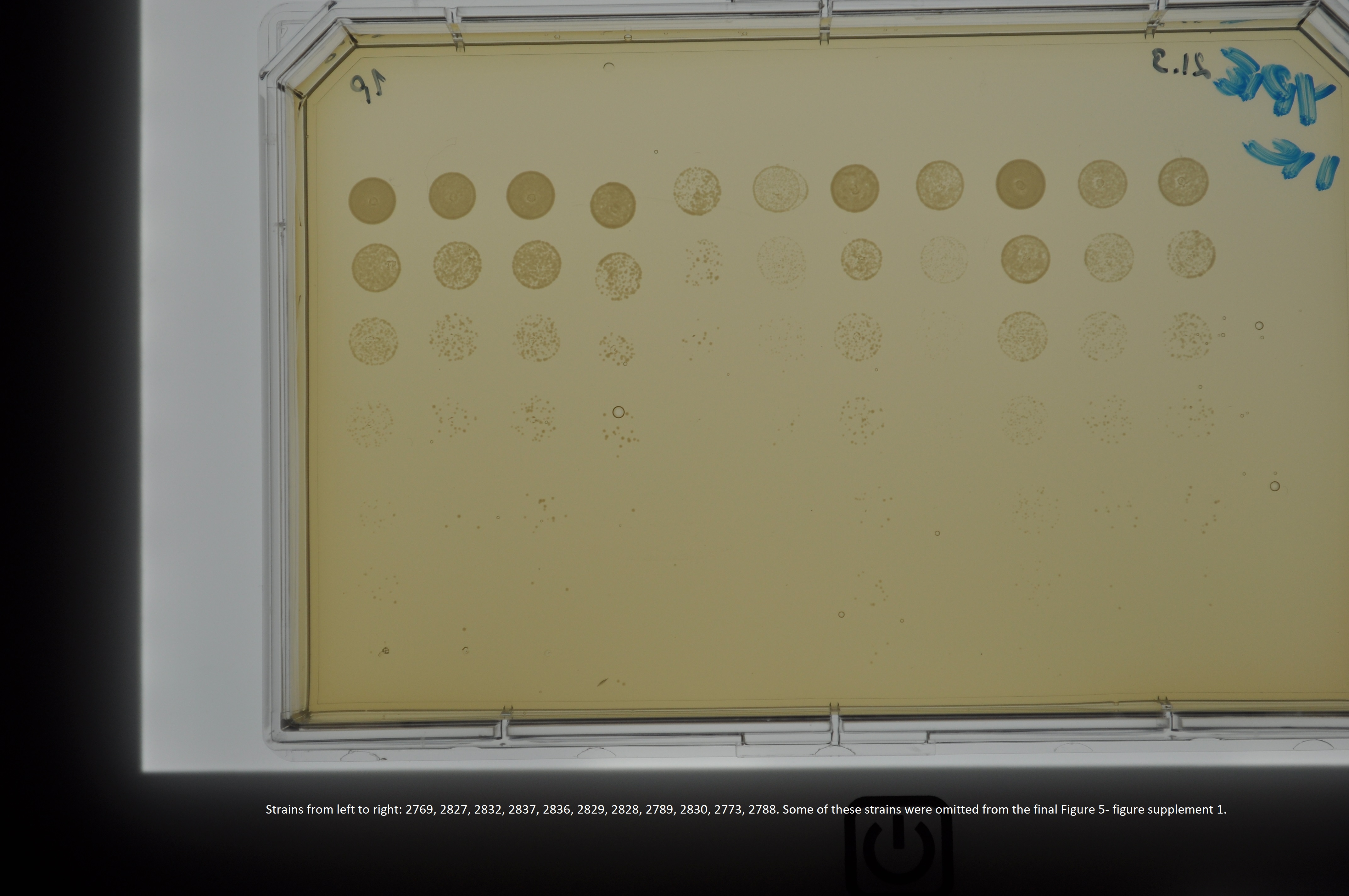

Supplement: Figure 5—figure supplement 1—source data 1. [file elife-69549-fig5-figsupp1-data1.zip › Figure5- figure supplement 1- source data 1/YPE/YPE_1000atc.JPG]

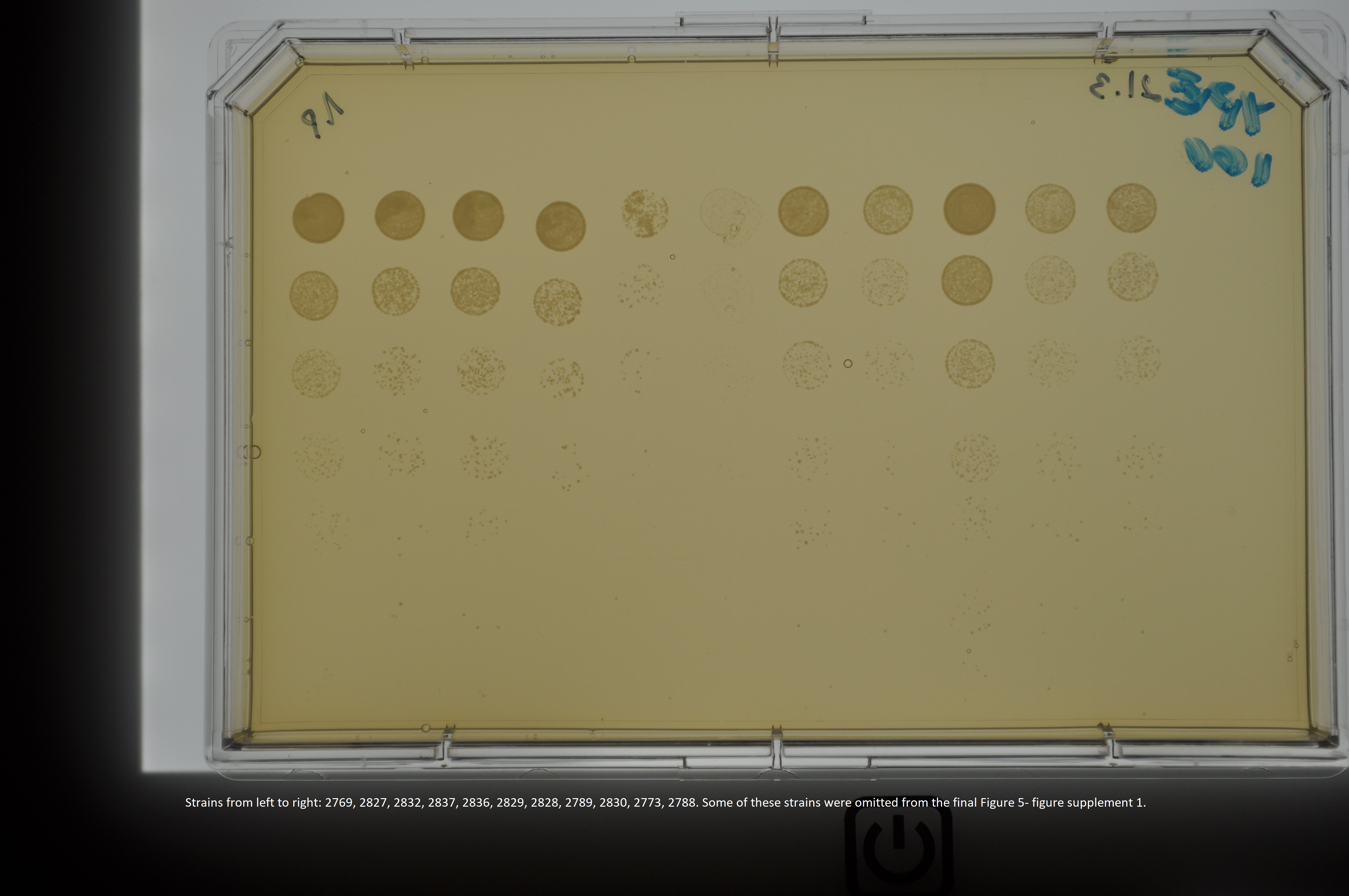

Supplement: Figure 5—figure supplement 1—source data 1. [file elife-69549-fig5-figsupp1-data1.zip › Figure5- figure supplement 1- source data 1/YPE/YPE_100atc.JPG]

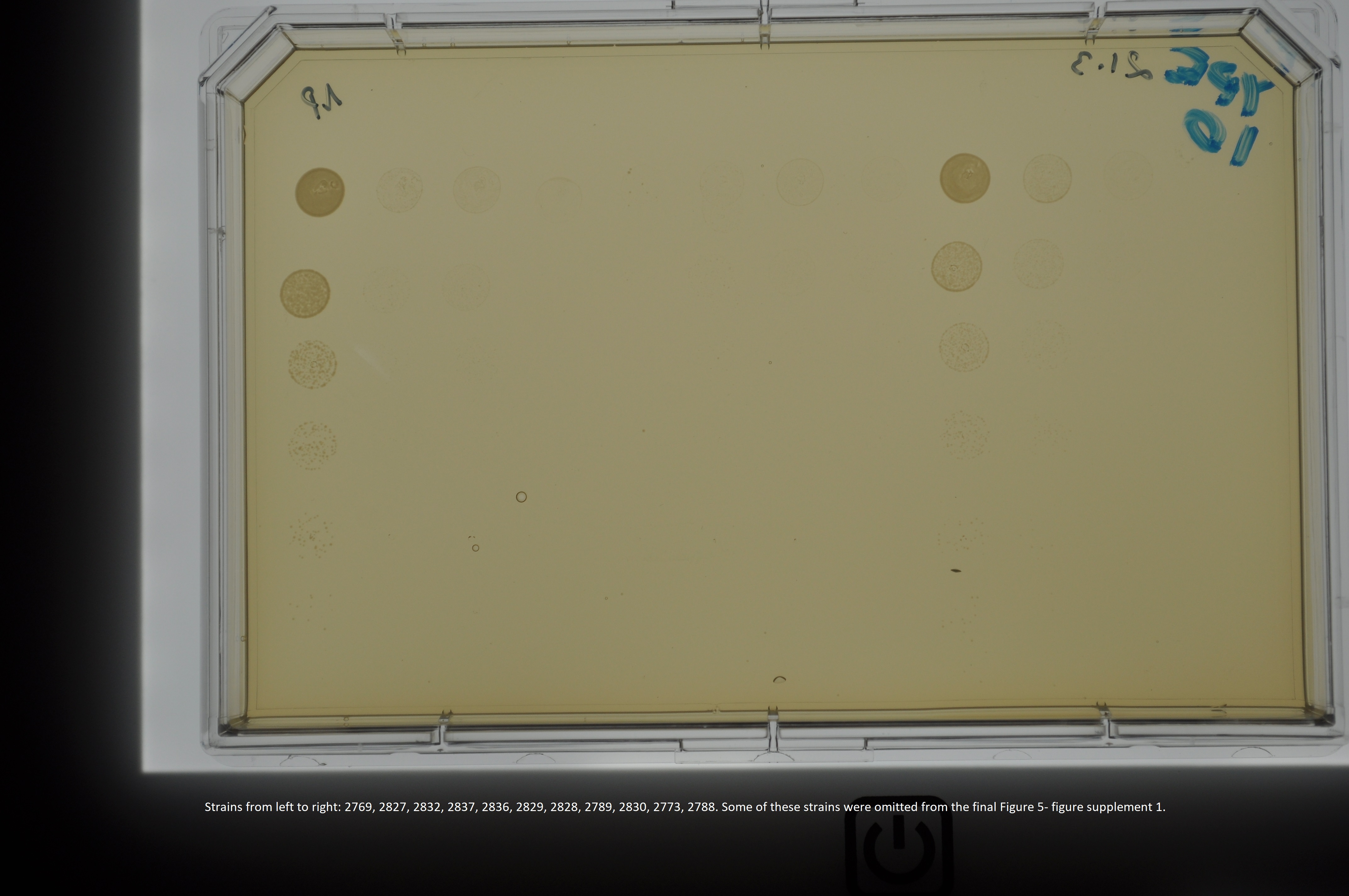

Supplement: Figure 5—figure supplement 1—source data 1. [file elife-69549-fig5-figsupp1-data1.zip › Figure5- figure supplement 1- source data 1/YPE/YPE_10atc.JPG]
